# Cross-Species Dissemination of Pandrug-Resistant *Acinetobacter baumannii* in Humans and Poultry in Egypt: Unveiling Shared Clones, Resistance Mechanisms, and Severe Clinical Outcomes

**DOI:** 10.3390/microorganisms14071409

**Published:** 2026-06-26

**Authors:** Azza S. El-Demerdash, Samah Eid, Rihaf Alfaraj, Nayera M. Al Atfeehy, Nissreen E. ElBadawy, Gehan K. Saleh, Neveen R. Bakry, Heba Farouk, Emad Sakr, Rania M. S. El-Malt

**Affiliations:** 1 Department of Biotechnology, Agricultural Research Center (ARC), Animal Health Research Institute (AHRI), Zagazig 44516, Egypt; 2 Reference Laboratory for Veterinary Quality Control on Poultry Production, Agriculture Research Centre (ARC), Animal Health Research Institute (AHRI), Dokki 12618, Egypt; 3 Department of Pharmaceutics, College of Pharmacy, King Saud University, Riyadh 11451, Saudi Arabia; 4 Department of Medical Microbiology and Immunology, Faculty of Medicine, Zagazig University, Zagazig 44519, Egypt; 5 Department of Biochemistry, Agricultural Research Center (ARC), Animal Health Research Institute (AHRI), Mansoura 35511, Egypt; 6 Department of Animal Hygiene and Zoonoses, Faculty of Veterinary Medicine, University of Sadat City, Sadat City 32897, Egypt; 7 Department of Microbiology, Agricultural Research Center (ARC), Animal Health Research Institute (AHRI), Zagazig Branch, Zagazig 44516, Egypt; raniaelmalt@yahoo.com

**Keywords:** antimicrobial resistome, carbapenem-resistant *A. baumannii* (CRAB), multilocus sequence typing, host–pathogen interactions, One Health, zoonotic transmission

## Abstract

The emergence and global dissemination of pandrug-resistant (PDR) *Acinetobacter baumannii* represents a critical public health crisis. This One Health study provides comprehensive surveillance and molecular characterization of carbapenem-resistant, extensively drug-resistant (XDR), and PDR *A. baumannii* isolates isolated from hospitalized patients and diseased chickens/environment in Egypt. We investigated cross-species clinical and pathological impacts, characterized resistance genes, and analyzed potential transmission links. Of 145 samples, 48 *A. baumannii* isolates were identified. Resistance profiling revealed an alarming prevalence, with PDR (56.3%) being the dominant phenotype, followed by XDR (43.7%), all exhibiting high multiple antibiotic resistance (MAR) indices (≥0.67). Chickens and humans infected with PDR *A. baumannii* suffered from increased neutrophilia, anemia, elevated inflammatory markers (CRP and procalcitonin), renal and liver impairment, and upregulation of *MMP-9* and *IL-8* response genes. Molecular analysis showed that all PDR isolates co-harbored multiple carbapenemase genes, including Class D beta-lactamases (*bla_OXA-23_* (most prevalent), *bla_OXA-48_*, *bla_OXA-58_*, *bla_OXA-24_*) and Class B metallo-beta lactamase (*bla_VIM_*, *bla_IMP_*, *bla_NDM_*). A substantial proportion also carried *bla_KPC_* (44.4%) and the *carO* gene (81.48%). Genotyping using ERIC PCR and Multilocus Sequence Typing (MLST) identified a high diversity (23 ERIC types, DI = 0.986). Significantly, two ERIC types (ET19 and ET20) contained isolates from both human and chicken sources. MLST confirmed this interspecies correlation, with isolates from both hosts clustering into Sequence Types (STs) ST1410 and ST1828. These findings confirm the rapid and alarming spread of highly virulent, multi-carbapenemase-producing PDR *A. baumannii* strains across the human–animal interface in Egypt. The detection of shared STs between clinical and poultry isolates underscores a potential zoonotic or environmental transmission route, necessitating integrated One Health surveillance and urgent infection control interventions.

## 1. Introduction

*Acinetobacter baumannii* has emerged as a critical global nosocomial pathogen, frequently causing severe healthcare-associated infections such as ventilator-associated pneumonia (VAP), bloodstream infections, and opportunistic sepsis [1,2]. Classified as a top-priority pathogen by the World Health Organization (WHO), carbapenem-resistant *A. baumannii* (CRAB) poses a formidable public health threat due to its capacity to rapidly fix multidrug-resistant (MDR), extensively drug-resistant (XDR), and pandrug-resistant (PDR) phenotypes [3,4,5]. Carbapenem resistance in this species is primarily driven by the enzymatic production of Ambler Class D oxacillinases (*bla_OXA-23_*, *bla_OXA-193_*, *bla_OXA-48_*, *bla_OXA-58_*, *bla_OXA-24_*) and Class B metallo-β-lactamases (*bla_VIM_*, *bla_IMP_*, and *bla_NDM_*), which are frequently complemented by non-enzymatic mechanisms such as the loss or alteration of outer membrane porins like CarO [6,7].

Under a One Health framework, *A. baumannii* is increasingly recognized as a multi-reservoir pathogen bridging livestock, human clinical settings, and their shared environments [8,9]. While agricultural environments and food-producing animals—particularly poultry—have been identified as critical reservoirs for extended-spectrum beta-lactamase (ESBL)-producing Gram-negative bacteria [9,10], comprehensive surveillance simultaneously tracking CRAB and PDR isolates across hospitalized patients, diseased chickens, and their immediate surroundings within a shared geographic region remains scarce. Furthermore, the correlation between the extreme antibiotic resistance profiles of these isolates and the underlying comparative host immunopathological responses (such as systemic haematological shifts, inflammatory clinical biomarkers, and pro-inflammatory gene expressions like *IL-8* and *MMP-9*) in both human and animal hosts remains poorly defined.

To bridge this knowledge gap, this study investigated the cross-species dissemination of CRAB across a human–poultry–environment continuum within the Sharqia Governorate, Egypt (30.7° N, 31.63° E). We aimed to characterize their molecular resistome profiles, determine genomic relatedness via Enterobacterial Repetitive Intergenic Consensus-PCR (ERIC-PCR) and Multilocus Sequence Typing (MLST), and evaluate host-specific pathological impacts. By establishing these transmission dynamics and clinical links, these findings aim to provide actionable data to inform regional infection control and integrated antimicrobial stewardship strategies.

## 2. Materials and Methods

### 2.1. Ethical Statement

This study was conducted in accordance with the ethical guidelines of the Institutional Animal Care and Use Committee (IACUC) of the Faculty of Veterinary Medicine, Zagazig University, Egypt (Approval No. ZU-IACUC/2/F/274/2024). Written informed consent for participation was obtained from the owners of the farms to collect the poultry samples.

Human participants were included in the study using bacterial isolates obtained from residual clinical samples. The study was approved by the Research Ethics Committee of the Faculty of Medicine, Zagazig University, Egypt (Approval No. ZU-IRB/910/2024), which granted an expedited review and a waiver of individual patient informed consent for the use of these residual, de-identified clinical isolates. This waiver was granted because: (1) The samples were residual (left over after clinical diagnosis and patient care), (2) The isolates were fully re-identified and anonymized prior to use in the research, and (3) The research posed no more than minimal risk to the patients’ rights or welfare.

In our investigation, the original sample collection was solely used for animal and patient care, and antimicrobial susceptibility testing to ensure proper diagnosis and treatment. All subsequent research procedures were performed in compliance with the ARRIVE guidelines (for the animal component) and the local and institutional regulations regarding re-identified data use.

### 2.2. Sample Collection

One hundred forty-five samples were collected from Sharkia Governorate in the Nile Delta region of Egypt, geographically positioned at approximately 30.7° N latitude and 31.63° E longitude between September 2024 and July 2025. This specific geographic focus allowed for a concentrated One Health assessment of overlapping clinical and agricultural ecosystems. Poultry samples included cloacal swabs (*n* = 22), fecal samples (*n* = 9), and nasal swabs (*n* = 12) from diseased chickens, as well as environmental samples such as chicken house swabs (*n* = 10), equipment swabs (*n* = 4), and food and water samples (*n* = 5 each). Human samples comprised midstream urine (*n* = 31), blood (*n* = 24), nasopharyngeal swabs (*n* = 4), sputum (*n* = 3), stool (*n* = 7), wound swabs (*n* = 3), and throat swabs (*n* = 6) obtained from hospitalized patients in the intensive care unit (ICU) at Zagazig University Hospital. Additionally, blood samples were collected from subjects and centrifuged to obtain serum, which was stored at −20 °C for further biochemical and hematological investigations. All samples were promptly transported to the laboratory in an icebox for bacteriological examination.

### 2.3. Acinetobacter baumannii Isolation and Identification

*A. baumannii* were isolated and identified using standard microbiological techniques. Clinical, poultry, and environmental samples were aseptically collected and inoculated onto selective MacConkey agar (Oxoid, Hampshire, UK). Isolates were further identified using biochemical tests (oxidase, catalase, indole, methyl red, Voges-Proskauer, citrate, and carbohydrate fermentation) and confirmed using the API 20NE system, as previously described by Peleg et al. [11]. DNA extraction from *A. baumannii* isolates was performed using the QIAamp DNA Mini Kit (Qiagen, Hilden, Germany) according to the manufacturer’s instructions. DNA purity and concentration were measured using a Nanodrop 2000/2000c spectrophotometer (Thermo Fisher Scientific, Cambridge, MA, USA).

Given reported difficulties in the definitive diagnosis of *A. baumannii* using *16S rRNA* alone, a rigorous, two-step molecular confirmation process was employed to ensure species specificity:

1. Conventional PCR Screening: All phenotypically identified isolates were initially screened by conventional PCR targeting the highly conserved *A. baumannii* species-specific 16S rRNA gene [12] (Table S1).
2. Real-Time PCR Confirmation: for definitive molecular identification, we performed a highly specific Real-time PCR (qRT-PCR) assay. This assay targeted three distinct *A. baumannii* species-specific genes: *rpoB*, *rpoD*, *fabD* [13] (Table S1). Reactions were carried out on a StepOne Real-Time PCR System using QuantiFast^®^ SYBR^®^ Green PCR Kit as per the manufacturer’s guidelines and under specific conditions as previously mentioned.

### 2.4. Antimicrobial Susceptibility Testing

The antimicrobial susceptibility test of *A. baumannii* isolates was investigated on Mueller–Hinton agar media (Oxoid, Hampshire, UK) utilizing the standard disc diffusion [14], and broth microdilution methods as per the regulations of the Clinical and Laboratory Standards Institute (CLSI) [15]. A panel of 15 standard antimicrobial discs (Oxoid, Hampshire, UK) within relevant 10 antimicrobial classes that were regularly used in human and veterinary medicine in Egypt were investigated including piperacillin/tazobactam (TZP, 110 µg), cefoxitin (FOX, 30 µg), cefepime (FEP, 30 µg), imipenem (IMP, 10 µg), meropenem (MEM, 10 µg), gentamicin (CN, 10 µg), amikacin (AK, 30 µg), tobramycin (TOB, 10 µg), ciprofloxacin (CIP, 5 µg), levofloxacin (LEV, 5 µg), sulfamethoxazole/trimethoprim (SXT, 25 µg), colistin (CT, 10 µg), tigecycline (TGC, 15 µg), tetracycline (TE, 30 µg), minocycline (MH, 30 µg). The interpretive measures of the CLSI were followed to categorize *Acinetobacter* isolates as resistant, intermediate, or susceptible [16]. Additionally, the minimum inhibitory concentrations (MICs) of the tested antimicrobials (Sigma-Aldrich, Taufkirchen, Germany) were detected using the broth microdilution technique following the guidelines of the CLSI [15]. For general susceptibility testing, *A. baumannii* ATCC 19606 was utilized as the standard quality control (QC) strain. To ensure the reliability of the critical colistin results, all colistin MICs were rigorously retested using the BMD method with *Escherichia coli* ATCC 25922 as an additional QC strain. MICs results for all drugs were interpreted according to CLSI breakpoints. MIC_90_ and MIC_50_ of the tested antimicrobials were determined as the lowest concentrations, which inhibit 90% and 50% of the tested *A. baumannii* isolates, respectively [17]. Multiple antimicrobial resistance (MAR) indices were calculated utilizing the following formula: MAR = a/b, where a denotes the number of antimicrobials to which the tested isolate was resistant, and b denotes the total number of tested antimicrobials [18].

Isolates were categorized according to standardized international consensus criteria [19] as follows: multidrug-resistant (MDR) if non-susceptible to three or more antimicrobial classes; extensively drug-resistant (XDR) if non-susceptible to all but two or fewer antimicrobial classes; and pandrug-resistant (PDR) if non-susceptible to all agents across all tested antimicrobial classes.

### 2.5. Investigation of Hematological and Biochemical Parameters

Hematological and biochemical parameters were measured in the collected blood samples using standard laboratory methods as described by Rifai [20]. Kidney function tests (creatinine and urea), total protein, glucose, C-reactive protein (CRP), procalcitonin (PCT), albumin, globulin, and cholesterol levels were analyzed using automated analyzers (Beckman Coulter AU5800 and Cobas e 411). The albumin-to-globulin (A/G) ratio was calculated. Complete blood count (CBC) parameters, including white blood cell (WBC) count, neutrophil count, red blood cell (RBC) count, packed cell volume (PCV), hemoglobin (Hb), and mean corpuscular hemoglobin (MCH), were determined using an automated hematology analyzer.

### 2.6. Gene Expression Analysis of Inflammation-Related Genes Among Acinetobacter baumannii Isolates from Diseased Chickens and Hospitalized Patients

Serum samples from *A. baumannii*-infected diseased chickens and hospitalized patients, as well as negative cases, were analyzed to determine the expression levels of host inflammation-related genes (*IL-8* and *MMP-9*) using quantitative real-time PCR (qRT-PCR). RNA was extracted from the serum samples using the QIAamp RNeasy Mini Kit (Qiagen, Hilden, Germany), followed by on-column DNase digestion to ensure the removal of genomic DNA contamination.

New primers, designed using Primer3 and FastPCR software, were optimized and validated experimentally. Primer sequences are listed in Table S1. The qRT-PCR reactions were performed, in triplicate, using 2× HERA SYBR^®^ Green RT-qPCR Master Mix (Willowfort, Nottingham, UK) following the manufacturer’s instructions. Reactions were carried out on a StepOne Real-Time PCR System under specific conditions listed in Table S1. Relative gene expression levels were calculated using the 2^−ΔΔCt^ method, with *β-actin* as the reference gene, as described by Yuan et al. [21] and El-Demerdash et al. [22].

### 2.7. Molecular Confirmation of Carbapenem Resistance in the Recovered Acinetobacter baumannii Isolates

Plasmid DNA was extracted using the Plasmid DNA Miniprep Kit (Thermo Fisher Scientific, Cambridge, MA, USA). DNA purity and concentration were measured using a Nanodrop 2000/2000c spectrophotometer (Thermo Fisher Scientific, Cambridge, MA, USA).

The presence of genes encoding carbapenemases was detected using established uniplex PCR assays.

- Target Genes: Uniplex PCR was employed to class D β-lactamase genes (*bla_OXA-23_*, *bla_OXA-48_*, *bla_OXA-58_*, and *bla_OXA-24_*), class B MBLs genes (*bla_VIM_*, *bla_IMP_*, and *bla_NDM_*), class A β-lactamase gene (*bla_KPC_*) and the carbapenem resistance-associated gene (*carO*).
- Primer Design and Validation: The primers for *bla_KPC_* and *carO* were designed *de novo* using Primer3 and FastPCR software. These newly designed primer products were validated by Sanger sequencing and alignment against the NCBI BLAST database to ensure target specificity.

PCR conditions were performed on a PTC-100 programmable thermal cycler (Peltier-Effect cycling, Staffordshire, UK) using Emerald Amp GT PCR master mix (Takara, San Jose, CA, USA). Primer sequences are listed in Table S1. PCR products were visualized on a 1.5% agarose gel stained with ethidium bromide.

To ensure reliability, several quality control measures were implemented: *A. baumannii* ATCC 19606 was used as the positive control for the detection of all ARGs, and representative amplicons for each target gene were confirmed via Sanger sequencing to ensure accuracy.

### 2.8. ERIC Genotyping

ERIC-PCR was employed as an initial genotyping method for the investigation of fingerprinting profiles of PDR and carbapenem-resistant *A. baumannii* isolates from various sources, utilizing the ERIC-F and ERIC-R primers (Table S1), as previously reported [23]. The factoextra package [24] in R software version 4.3.3 (R Project for Statistical Computing, https://www.r-project.org/, accessed on 1 May 2026) was used for analyzing the ERIC-PCR band patterns. The Dice coefficient was used to assess the similarity of the fingerprinting profiles, and a dendrogram was generated employing the unweighted pair group technique with arithmetic mean (UPGMA). The ERIC-PCR discriminatory index (*D* values) was determined using Simpson’s index of diversity [25]. A *D* value exceeding 0.9 indicates effective discrimination.

### 2.9. Multilocus Sequence Typing (MLST)

MLST was applied to chicken and human *A. baumannii* isolates that shared the same ERIC type (ET) to confirm a potential correlation between human and chicken isolates. The MLST was performed using the Pasteur scheme [26] according to the techniques outlined in the Institut Pasteur MLST databases (http://pubmlst.org/abaumannii/, accessed on 1 January 2026). The Pasteur scheme is intended to detect the following seven housekeeping genes: *cpn60* (60-KDa chaperonin), *fusA* (elongation factor EF-G), *gltA* (citrate synthase), *pyrG* (CTP synthase), *recA* (homologous recombination factor), *rplB* (50S ribosomal protein L2), and *rpoB* (RNA polymerase subunit B). Each isolate was allocated an allele number for each gene, and the 7 allele numbers were aggregated to produce a specific sequence type (ST) using the pubMLST database (https://pubmlst.org/abaumannii/, accessed on 1 January 2026) [27].

### 2.10. Statistical Data Analysis

Categorical variables, such as the prevalence of *A. baumannii* from different sources and antimicrobial resistance profiles, were analyzed using the Chi-square test. Numerical variables were compared using the independent-samples *t*-test. Statistical significance was set at *p* < 0.05. Analyses were performed with SPSS version 26 (IBM Corp., Armonk, NY, USA).

For data visualization, R software version 4.3.3 ((R Project for Statistical Computing, https://www.r-project.org/, accessed on 1 May 2026) was used. The prevalence of *A. baumannii* and the frequencies of the analyzed resistance phenotypes/genes were represented as stacked bar graphs, where subcolumns are part of the total column, and boxplots utilizing the ggplot package in R software version 4.3.3 [28]. Hierarchical clustering (HA) heatmaps, HA dendrogram, and principal component analysis (PCA) biplots were employed to explore the overall distribution of parameters in the isolates and their relationships, utilizing the pheatmap [29] and factoextra [24] packages in R software version 4.3.3. Additionally, random forest classification was used to identify the most significant variables for predicting *A. baumannii* occurrence utilizing the randomForest package in R software version 4.3.3 [30]. Before conducting correlation studies, variables were assessed for normality with a Q–Q plot. The Pearson correlation (*r*) was calculated and illustrated utilizing the Hmisc package in R software version 4.3.1 [31]. Substantial correlation pairs were identified at a *p*-value of 0.05. GraphPad Prism version 8 (San Diego, CA, USA) was also used for graphical illustration of hematological and biochemical parameters of diseased chickens and humans infected with *A. baumannii* isolates belonging to various resistance categories.

## 3. Results

### 3.1. Clinical Manifestations of Diseased Chickens and Hospitalized Patients

The majority of diseased chickens (72.1%) exhibited general intestinal/diarrheal symptoms, including diarrhea, decreased appetite, weight loss, abdominal distension, and dehydration. Respiratory signs, such as nasal discharge, coughing, and difficulty breathing, were observed in 20.8% of infected birds. Reduced egg production accompanied by severe respiratory manifestations was noted in 13.9% of the infected chickens.

In human patients, isolates were primarily recovered from patients presenting with urinary tract infection (UTI)-like symptoms/bacteriuria (39.7%) and bloodstream infection (25.6%). Patients presenting with gastrointestinal symptoms (including diarrhea, cramping, nausea, vomiting, and fever) accounted for 16.7% of the study population, with 2.6% experiencing severe symptoms (including bloody stools). Isolates from pyogenic wound infections were observed in 3.8% of patients. Respiratory tract manifestations, such as coughing, shortness of breath, and chest pain, were detected in 11.5% of patients, with 8.9% experiencing severe respiratory manifestations (VAP), including fever and sputum production (Table 1).

### 3.2. Prevalence of Acinetobacter baumannii in Diseased Chickens, Their Environment, and Hospitalized Patients in the Study Area

Of the 145 samples collected from Sharkia Governorate, Egypt, 48 (32%) were identified as *A. baumannii* based on conventional identification techniques (Supplementary Figure S1 and Table S2). All 48 retrieved *A. baumannii* isolates harbored the *A. baumannii* species-specific *16S rRNA* gene. Additionally, the Real-time PCR assay showed that all 48 obtained isolates harbored *A. baumannii* species-specific *rpoB*, *rpoD*, and *fabD* genes with Ct values of 25.79 ± 0.31, 26.26 ± 0.58, and 24.77 ± 0.62, respectively, and molecularly confirmed to be *A. baumannii. A. baumannii* was slightly more prevalent in chicken than human samples (37.2% and 35.9%, respectively), with lower prevalence in chicken environmental samples (16.7%). No significant difference was observed in the prevalence of *A. baumannii* among various sample sources (*p* = 0.19).

Regarding sample types, *A. baumannii* was most frequently isolated from human throat swabs (66.7%) and stool samples (57.1%), while no isolates were recovered from nasopharyngeal swabs. Among chicken samples, *A. baumannii* was more prevalent in fecal samples (55.6%) and cloacal swabs (45.5%), compared to nasal swabs (8.3%). In the poultry environmental samples, *A. baumannii* was most common in food samples (40%), followed by equipment (25%) and water samples (20%). No *A. baumannii* isolates were recovered from chicken house samples (Figure S2A). Overall, no statistically significant difference was observed in the prevalence of *A. baumannii* among various sample types (*p* > 0.05) (Supplementary Table S2).

Regarding the clinical conditions of infected hosts, the highest isolation rates in human patients were observed in those with gastrointestinal tract colonization (100%) and those with severe respiratory manifestations (VAP) (71.4%). Isolates were also recovered from patients presenting with UTI-like symptoms/bacteriuria (35.5%) and bloodstream infections (35%). For diseased chickens, *A. baumannii* was most frequently isolated from birds with intestinal/diarrheal symptoms (48.4%), followed by those with decreased egg production and severe respiratory manifestations (16.7%). No *A. baumannii* isolates were recovered from humans with simple respiratory manifestations or from chickens with only respiratory or severe respiratory manifestations (Figure S2B). No statistically significant difference was observed in the prevalence of *A. baumannii* among various clinical conditions (*p* > 0.05) (Supplementary Table S2).

**Figure 1:**
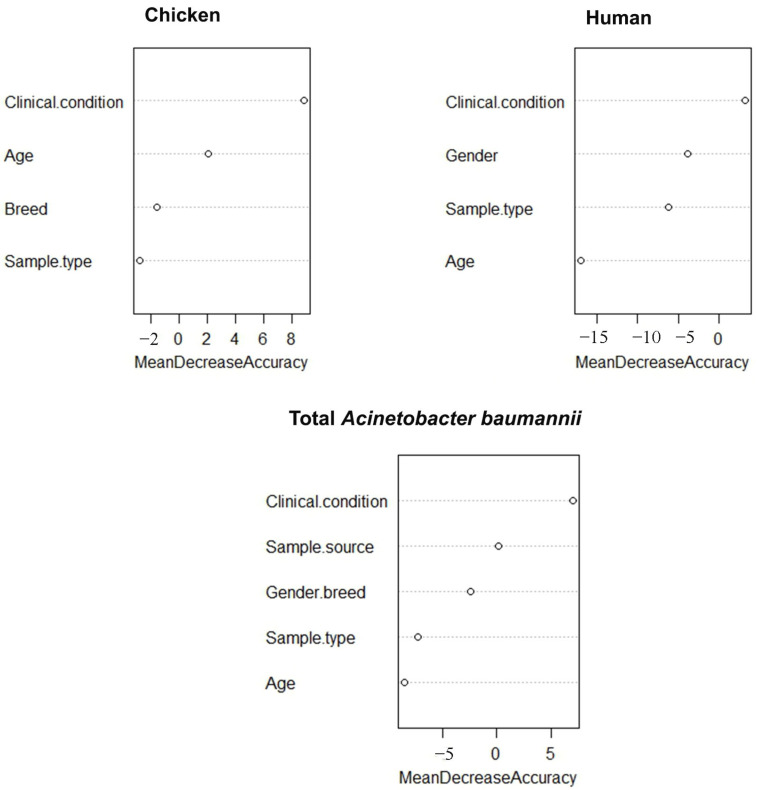
Random forest classification displaying ranked significance for each variable in predicting the prevalence of *Acinetobacter baumannii*. The X-axis denotes the mean decrease in accuracy of the classification when the respective variable was removed.

Clinical condition, sample source, gender/breed, sample type, and age were found to influence the prevalence of *A. baumannii* in various sample sources. The Random Forest classification showed that the clinical condition had the highest ranked significance among variables according to the mean decrease in accuracy of the classification when the respective variable was removed. Sample type and age had the least ranked significance among variables in chickens and humans, respectively (Figure 1). The univariate logistic regression analysis showed that breed is the most significant (*p* = 0.039) variable affecting the prevalence of *A. baumannii* in chickens, with broiler chickens being the most susceptible breed (Supplementary Table S3). In humans, gender and sample type were the most significant (*p* = 0.019 and 0.048) variables affecting the prevalence of *A. baumannii*, with males being more susceptible than females, and throat swabs showed the highest prevalence of *A. baumannii* (Supplementary Table S3).

### 3.3. Antimicrobial Susceptibility and Phenotypic Clustering

**Figure 2:**
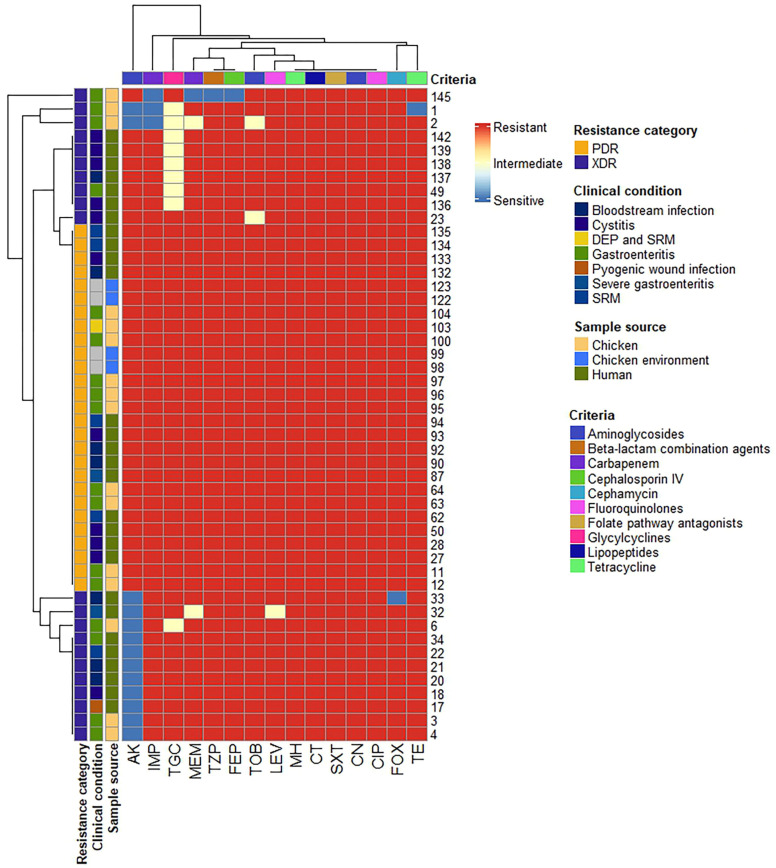
Hierarchical clustering heatmap depicting the distribution of antimicrobial susceptibility patterns among the recovered *Acinetobacter baumannii* isolates by broth microdilution assay. Various sample sources, clinical conditions, and antimicrobial classes are presented as color-coded. TZP: piperacillin/tazobactam; FOX: cefoxitin; FEP: cefepime; IMP: imipenem; MEM: meropenem; CN: gentamicin; AK: amikacin; TOB: tobramycin; CIP: ciprofloxacin; LEV: levofloxacin; SXT: sulfamethoxazole/trimethoprim; CT: colistin; TGC: tigecycline; TE: tetracycline; MH: minocycline; MDR: multidrug-resistant; XDR: extensively drug-resistant; PDR: pan drug-resistant.

Antimicrobial susceptibility testing of the 48 *A. baumannii* isolates revealed extensive, multi-class resistance phenotypes (Figure 2 and Supplementary Tables S4 and S5). Both disc diffusion and broth microdilution (BMD) methods demonstrated absolute resistance (100.0%) to gentamicin (MIC: 516–1024 μg/mL), ciprofloxacin, sulfamethoxazole/trimethoprim, and minocycline (MICs: 1024 μg/mL for the latter three). BMD confirmed universal colistin resistance with high minimum inhibitory concentrations (MIC: 64–1024 μg/mL; Supplementary Table S6), validated by compliant quality control ranges for *E. coli* ATCC 25922 (1 μg/mL) and *A. baumannii* ATCC 19606. BMD profiles correlated strongly with disc diffusion findings, exhibiting only minor variations for colistin (100.0% vs. 97.92%) and levofloxacin (97.92% vs. 95.83%). High resistance rates were also observed for piperacillin/tazobactam, cefepime, cefoxitin, tetracycline, levofloxacin (97.92% each), tobramycin (95.83%), imipenem, and meropenem (93.75% each), while amikacin (72.92%) and tigecycline (78.57%) displayed the lowest resistance frequencies.

Hierarchical cluster analysis resolved the 48 isolates into three primary branches spanning four distinct phenotypic clusters, with only seven lineages diverging from the dominant groups (Figure 2). Concurrently, clustering of the 15 tested antimicrobials revealed two primary branches: the first grouped co-resistance to cefepime and piperacillin/tazobactam, while the second clustered minocycline, colistin, sulfamethoxazole/trimethoprim, gentamicin, and ciprofloxacin together.

Stratification by collection source revealed a higher overall resistance burden in human strains, which exhibited absolute resistance (100.0%) to six critical agents: piperacillin/tazobactam, cefepime, imipenem, ciprofloxacin, colistin, and tetracycline. In contrast, poultry isolates demonstrated absolute resistance exclusively to levofloxacin. Source-dependent variations in resistance frequencies were statistically significant only for imipenem (*p* = 0.048; Supplementary Table S4). All collection sources showed advanced multi-class resistance patterns (Supplementary Figure S3 and Table S7); chicken isolates resisted 9 (18.75%), 10 (18.75%), or 11 (62.50%) drug classes, while human isolates resisted 9 (32.14%), 10 (21.43%), or 11 (46.43%) classes. Global collection profiles identified 43.75% (*n* = 21) of the isolates as extensively drug-resistant (XDR) and 56.25% (*n* = 27) as pandrug-resistant (PDR), the latter comprising 46.43% of human, 62.50% of chicken, and 100.0% of environmental strains. Multiple Antibiotic Resistance (MAR) indices reached high-risk thresholds across both poultry (≥0.67, resisting ≥ 10 agents) and human cohorts (≥0.80, resisting ≥ 12 agents).

### 3.4. Analysis of Hematological and Biochemical Parameters

Our findings reveal a significant and comparable systemic impact of *A. baumannii* infection on both chickens and human patients. Both groups exhibited similar physiological disturbances, including anemia (decreased hemoglobin (Hb), packed cell volume (PCV), and red blood cell (RBC) count, along with elevated Mean Corpuscular Hemoglobin (MCH), inflammation (leukocytosis, neutrophilia, and elevated C-reactive protein (CRP) and procalcitonin (PCT), and signs of impaired renal function (elevated creatinine and urea). Liver function showed consistent signs of disruption, marked by low albumin, elevated total protein and globulin, and a normal albumin/globulin ratio. Interestingly, hyperglycemia was present in the majority of infected chickens (81.25%), while human patients presented with a mixed glucose profile (32.1% hyperglycemia and 64.3% hypoglycemia). Furthermore, nearly all infected chickens (68.75%) had elevated cholesterol levels, a finding not mirrored in human patients, who maintained normal cholesterol levels (Table 2 and Supplementary Figure S4).

**Figure 3:**
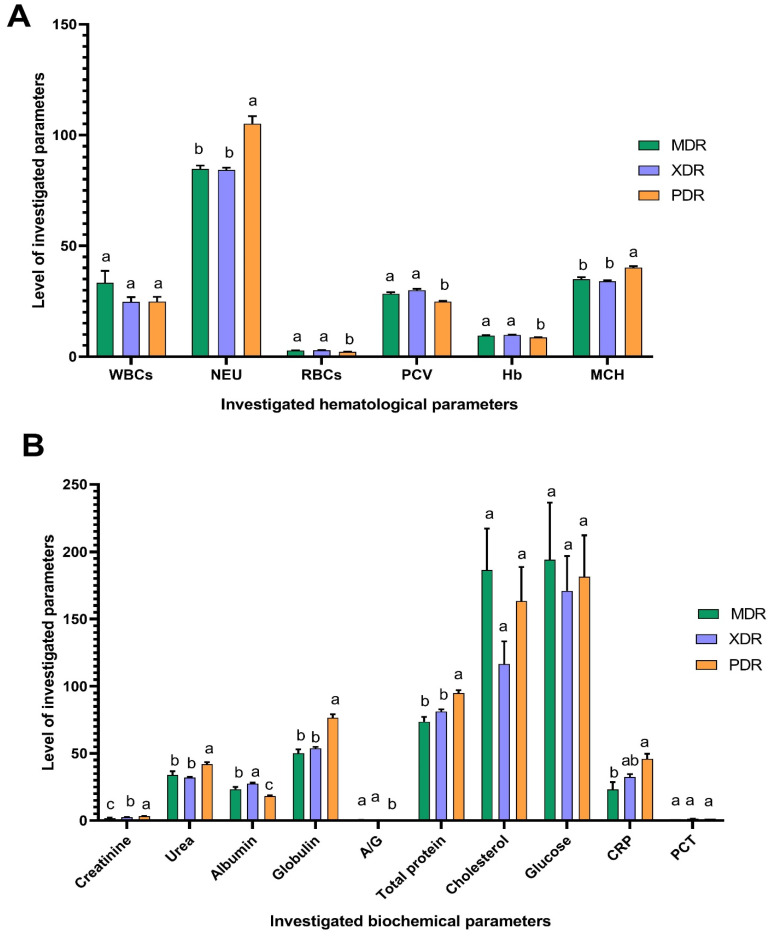
Analysis of hematological (**A**) and biochemical (**B**) parameters of diseased chickens, and humans infected with *Acinetobacter baumannii* isolates belonging to various resistance categories. WBCs: white blood cells; RBCs: red blood cells; PCV: packed cell volume; Hb: hemoglobin; MCH: mean corpuscular hemoglobin; NEU: neutrophils; CRP: C-reactive protein; PCT: procalcitonin; A/G: albumin/globulin ratio; MDR: multidrug-resistant; XDR: extensively drug-resistant; PDR: pandrug-resistant. ^a–c^ columns with various superscript letters denote statistical significance at *p* < 0.05. The measuring unit of each parameter is illustrated in Table S7.

The severity of the physiological changes correlated with the resistance category of the *A. baumannii* isolate. Infections caused by PDR isolates were consistently associated with the most severe clinical pathology: they induced the lowest levels of Hb, PCV, RBCs, and albumin, and the highest levels of CRP, PCT, neutrophils, MCH, urea, creatinine, total protein, and globulin (Figure 3). Statistical analysis confirmed highly significant differences in the levels of neutrophils, RBCs, PCV, Hb, MCH, urea, creatinine, total protein, albumin, globulin, A/G ratio, and PCT (*p* < 0.0001 for each), as well as CRP (*p* = 0.002), among infections caused by PDR, XDR, and MDR isolates. However, differences in blood glucose, WBCs, and cholesterol levels were not statistically significant across these resistance categories. Separately, when comparing hospitalized patients by various clinical conditions, all of the investigated parameters showed no significant differences (*p* > 0.05), with the exception of urea (*p* = 0.021).

When directly comparing the hosts, diseased chickens exhibited higher levels of MCH, blood glucose, urea, and cholesterol, alongside lower levels of Hb, PCV, and RBCs, compared to hospitalized human patients. Conversely, CRP levels were significantly higher in humans than in chickens (Supplementary Figure S5). This host-specific difference was statistically significant for blood glucose, CRP, WBCs, PCV, RBCs, MCH, cholesterol (all *p* < 0.0001), urea (*p* = 0.032), and albumin (*p* = 0.018). No significant inter-host differences were found for PCT, neutrophils, Hb, creatinine, globulin, A/G ratio, or total protein (Table 2).

### 3.5. Gene Expression Analysis of Inflammation-Related Genes

**Figure 4:**
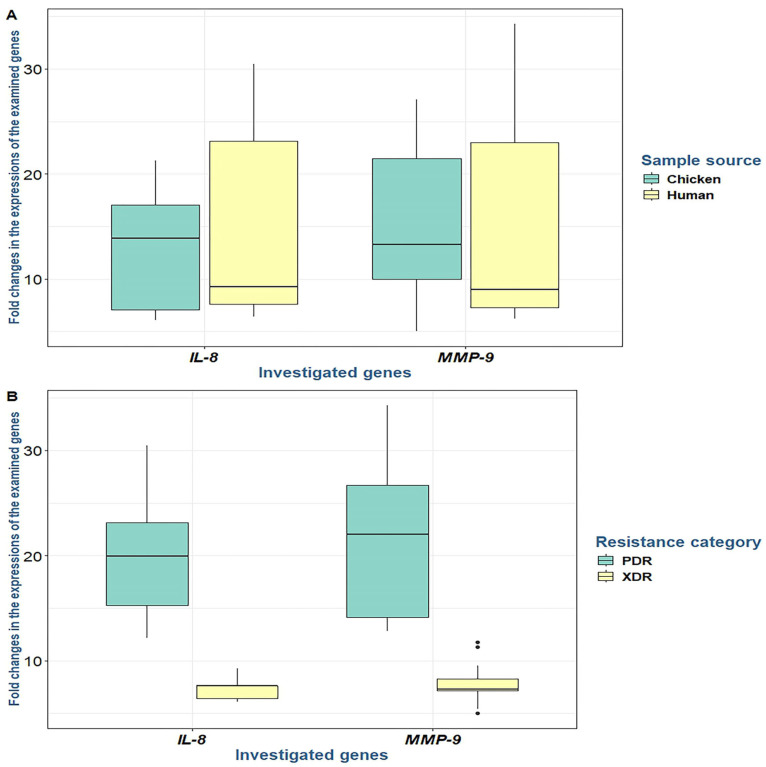
Relative mRNA expression levels of interleukine-8 (*IL-8*) and matrix metalloproteinases-9 (*MMP-9*) genes among *Acinetobacter baumannii* isolates belonging to various resistance categories (**A**) and sources (**B**). MDR: multidrug-resistant; XDR: extensively drug-resistant; PDR: pandrug-resistant.

Interestingly, the expression levels of *IL-8* and *MMP-9*, key inflammatory genes, were significantly upregulated in *A. baumannii* isolates from both diseased chickens and hospitalized patients, suggesting their role in enhancing neutrophil migration and promoting inflammation. The PDR *A. baumannii* isolates exhibited significantly higher mRNA transcription levels of *MMP-9* (up to 34.3-fold) and *IL-8* (up to 30.5-fold) compared to XDR isolates (*p* < 0.05) (Figure 4A).

Human *A. baumannii* isolates exhibited significantly higher mRNA transcription levels of *MMP-9* (up to 34.3-fold) and *IL-8* (up to 30.5-fold) compared to chicken isolates (up to 27-fold and 21-fold, respectively) (Figure 4B). No significant differences were observed in the transcription levels of *IL-8* and *MMP-9* among *A. baumannii* isolates from different origins (*p* = 0.552 and 0.872, respectively).

### 3.6. Distribution of Carbapenemase Genes Among PDR Acinetobacter baumannii Isolates

**Figure 5:**
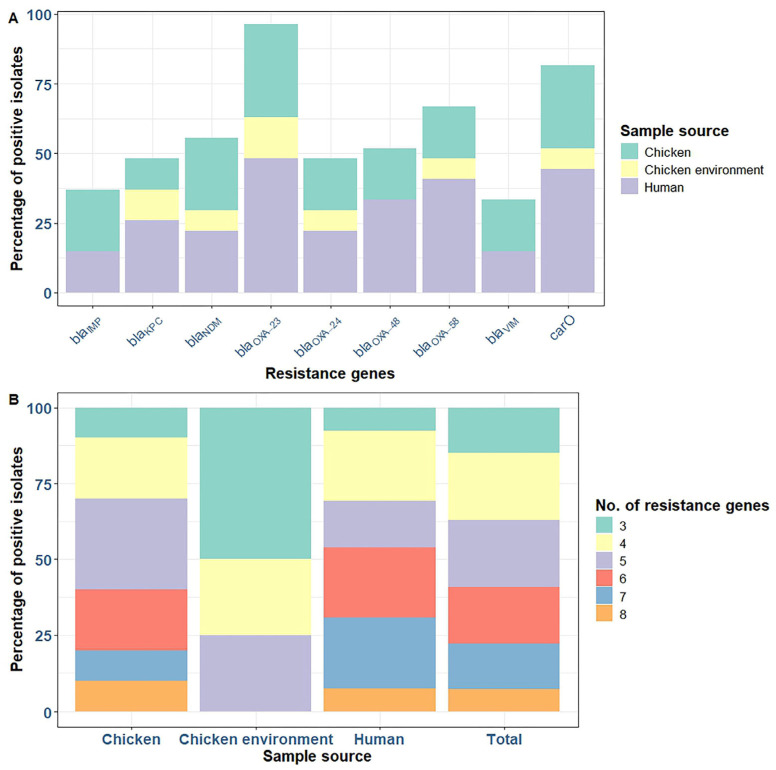
Stacked bar graph illustrating the prevalence (**A**) and distribution (**B**) of carbapenemase genes among PDR *Acinetobacter baumannii* isolates from diseased chickens, their environment, and hospitalized patients. In the stacked bar plot, the frequency was calculated concerning the total number of examined isolates (*n* = 27) for each parameter, and sub-columns are calculated as a part of the total column.

All 27 PDR and carbapenem-resistant *A. baumannii* isolates harbored at least one carbapenemase gene. The most prevalent carbapenemase genes were *bla_OXA-23_* (96.29%), *bla_OXA-58_* (66.67%), *_blaOXA-48_* (51.85%), and *bla_OXA-24_* (48.15%). Additionally, 21 (77.78%) isolates produced MBLs, including *bla_NDM_* (55.56%), *bla_IMP_* (37.04%), and *bla_VIM_* (33.33%). Thirteen isolates (48.15%) harbored the class A carbapenemase gene, *bla_KPC_*. Furthermore, 22 isolates (81.48%) carried the carbapenem resistance-associated gene, *carO*. Notably, all PDR CRAB isolates possessed a combination of both OXA-type and MBLs carbapenemase genes. Twelve isolates (44.4%) harbored class A, B, and C β-lactamase genes (Figure 5A). There were no significant differences in the prevalence of *bla_KPC_*, *bla_VIM_*, *bla_IMP_*, *bla_NDM_*, *bla_OXA-23_*, *bla_OXA-48_*, *bla_OXA-58_*, *bla_OXA-24_*, and *carO* genes among CRAB isolates from different sources (*p* = 0.287, 0.25, 0.099, 0.581, 0.519, 0.053, 0.212, 1, and 0.185, respectively). Twenty-four different resistance gene profiles were identified among the PDR CRAB isolates. The most common profiles included *bla_KPC_*, *bla_NDM_*, *bla_OXA-23_*; *bla_OXA-23_*, *bla_OXA-48_*, *bla_OXA-58_*; and *bla_OXA-23_*, *bla_OXA-58_*, *bla_OXA-24_*, *carO* (7.4% each).

Regarding the source of isolation, *bla_NDM_*, *bla_IMP_*, and *bla_VIM_* genes were more prevalent in chicken-derived CRAB isolates (70%, 60%, and 50%, respectively) compared to human and environmental isolates. In contrast, *carO*, *bla_OXA-58_*, *bla_OXA-48_*, and *bla_KPC_* genes were more prevalent in CRAB isolates from hospitalized patients (92.31%, 84.62%, 69.23%, and 53.85%, respectively) compared to chicken and environmental isolates (Figure 5A).

Interestingly, all PDR CRAB isolates carried at least three carbapenemase genes. Chicken environmental isolates harbored three, four, or five carbapenemase genes (50%, 25%, and 25%, respectively), while none carried *bla_OXA-48_*, *bla_IMP_*, or *bla_VIM_* genes. Notably, all human and chicken environmental isolates were positive for the *bla_OXA-23_* gene (Figure 5B). One human isolate (7.69%) and one chicken isolate (10%) possessed all eight investigated carbapenemase genes. Additionally, four (14.81%) and five (18.52%) isolates harbored seven and six carbapenemase genes, respectively (Figure 5B).

### 3.7. Correlation Analysis Between PDR Acinetobacter baumannii’s Carbapenemase Genes, Hematological, and Biochemical Parameters, and Expression Levels of IL-8 and MMP-9 Genes

**Figure 6:**
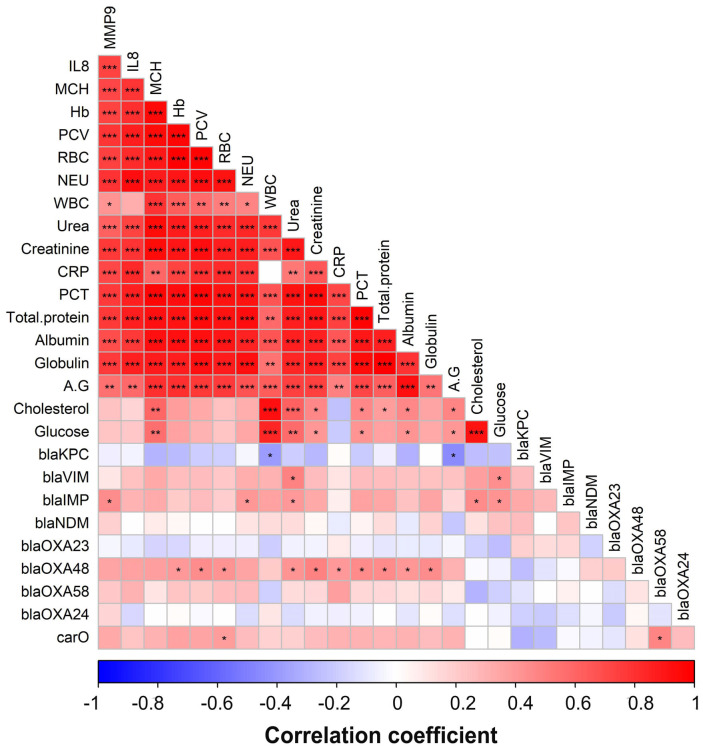
Pairwise correlation (*r*) among the investigated carbapenemase genes, hematological, and biochemical markers, and the transcription of *IL-8* and *MMP-9* genes. Blue and red colors imply negative and positive correlations, respectively. The darker colors denote stronger negative or positive correlations. Stars refer to significant correlations; * *p* < 0.05, ** *p* < 0.01, *** *p* < 0.001. WBC: white blood cells; RBC: red blood cells; PCV: packed cell volume; Hb: hemoglobin; MCH: mean corpuscular hemoglobin; NEU: neutrophils; CRP: C-reactive protein; PCT: procalcitonin; A/G: albumin/globulin ratio.

Correlation analysis between antimicrobial resistance genotypes and host hematological and biochemical parameters, and expression levels of *IL-8* and *MMP-9* genes can help fill several biological landscapes related to host–pathogen interactions, infection severity, and treatment outcomes. Hierarchical clustering analysis of carbapenemase genes, hematological and biochemical parameters, and the expression of *IL-8* and *MMP-9* genes revealed seven branches and one cluster. Hematological and biochemical parameters, along with *IL-8* and *MMP-9* gene expression, formed a distinct cluster (Supplementary Figure S6A). Pairwise correlation analysis further elucidated these relationships (Figure 6). The *carO* gene was significantly and positively correlated with *bla_OXA-58_* and RBCs (*r* = 0.47 and 0.38, respectively, *p* < 0.05). The *bla_OXA-48_* gene was significantly and positively correlated with Hb, CRP, urea, albumin, PCT, total protein, globulin, PCV, RBCs, and creatinine (*r* = 0.39, 0.41, 0.41, 0.45, 0.45, 0.45, 0.44, 0.43, and 0.48, respectively, *p* < 0.05). The *bla_IMP_* gene was significantly and positively correlated with *MMP-9* gene expression, neutrophils, urea, cholesterol, and glucose (*r* = 0.45, 0.41, 0.39, 0.46, and 0.43, respectively, *p* < 0.05) (Figure 6).

The *bla_VIM_* gene was significantly and positively correlated with urea and glucose (*r* = 0.48 and 0.44, respectively, *p* < 0.05). In contrast, the *bla_KPC_* gene was significantly and negatively correlated with WBCs and the A/G ratio (*r* = −0.39 and −0.45, respectively, *p* < 0.05). A strong positive correlation was observed between the expression of *IL-8* and *MMP-9* genes (*r* = 0.73, *p* < 0.0001). Both genes were significantly and positively correlated with MCH, Hb, RBCs, urea, creatinine, CRP, total protein, albumin, globulin, and A/G (*r* = 0.84–0.56, *p* < 0.05). *IL-8* was also significantly and positively correlated with neutrophils (*r* = 0.94) and PCT (*r* = 0.86), while *MMP-9* was significantly and positively correlated with PCT, neutrophils, and WBCs (*r* = 0.76, 0.79, and 0.42, respectively, *p* < 0.05). A strong positive correlation was also observed between PCT and CRP (*r* = 0.71, *p* < 0.0001). Overall, a general positive correlation was observed among most hematological and biochemical parameters (Figure 6).

The correlation between PDR *A. baumannii* isolates based on their carbapenemase gene profiles, hematological and biochemical markers, and *IL-8* and *MMP-9* gene expression is depicted in Supplementary Figures S6 and S7. Hierarchical clustering analysis revealed two main branches, one containing chicken environmental isolates and the other containing both chicken and human isolates, and two clusters. Interestingly, most PDR isolates exhibited unique profiles, with only two human and two chicken environmental isolates sharing similar characteristics (Supplementary Figure S6B). The PCA (Principal component analysis) biplot further supported these findings, demonstrating specific clustering patterns for PDR isolates from various sources (Figure S7).

### 3.8. Genotyping and Epidemiological Association of PDR Acinetobacter baumannii Poultry and Human Isolates via ERIC-PCR, and MLST Techniques

**Figure 7:**
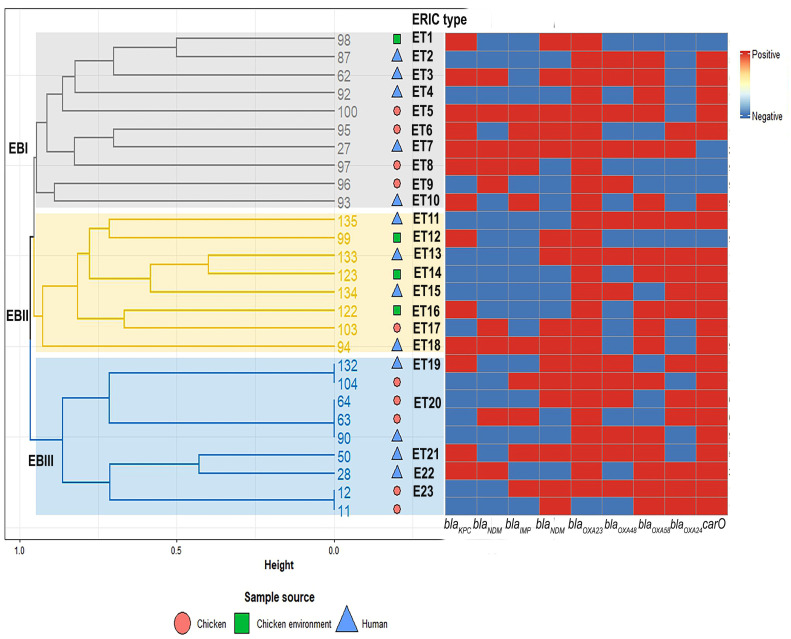
Hierarchical clustering dendrogram revealing ERIC-PCR genotyping and heatmap depicting the distribution of carbapenemase genes among PDR *A. baumannii* isolates from diseased chickens, their environment, and hospitalized humans in the research area. EB: ERIC branch; ET: ERIC type.

The typing and genetic linkages among the 27 PDR and carbapenem-resistant *A. baumannii* isolates were examined using ERIC-PCR. This link can be identified by the banding patterns produced with ERIC primers. Figure 7 illustrates that ERIC genotyping categorized 27 CRAB isolates into three ERIC branches (EB): EBI, EBII, and EBIII, comprising 10, 8, and 9 isolates, respectively, with three clusters. The EBs comprised 23 unique ERIC types (ET) with band widths varying from 369 to 1352 bp and a discrimination score (*D*) of 0.986, indicating that ERIC-PCR genotyping was successful in distinguishing the examined isolates. Three ETs (ET19, ET20, and ET23) exhibited clusters including two, three, and two identical isolates for each ET, respectively (Figure 7). The clustering of human and chicken *A. baumannii* isolates, as observed in ET19 and ET20, revealed the possible genetic relatedness of the isolates from both sources (Figure 7). The Dice coefficient similarity matrix for chicken and human isolates exhibiting identical profiles was 100% for ET19 and ET20.

MLST of the five PDR and carbapenem-resistant *A. baumannii* isolates belonging to ET19 and ET20 revealed two STs (ST1410, ST1828), which confirmed the results of ERIC-PCR (Table 3). MLST analysis showed that two *A. baumannii* isolates from human and chicken sources (Code no. Hu132, Ch104) clustered together in ST1410. Additionally, one human and two chicken *A. baumannii* isolates (Code no. Hu90, Ch63, Ch64) were clustered together in ST1828. ST1410 and ST1828 shared three allelic profiles, with four locus variants (Table 3). MLST analysis showed genetic relatedness of *A. baumannii* isolates from human and chicken origins with a potential cross-species link.

## 4. Discussion

*A. baumannii* is a serious global nosocomial pathogen [32]. Its widespread isolation in healthcare settings [33,34] and survival in various animal sources [35] mandate a coordinated One Health response, especially given the alarming rise of CRAB and PDR strains [34,36]. To our knowledge, this is the first study to directly compare PDR CRAB isolates obtained from diseased chickens, their environment, and hospitalized patients within the same Egyptian governorate, simultaneously characterizing the molecular mechanisms and clinical impact in both human and animal hosts. Our investigation found comparable prevalence rates in diseased chickens (37.2%) and hospitalized humans (35.9%), with lower rates in the poultry environment (16.7%). The human prevalence rate aligns with recent reports from Pakistan, Turkey, and Malaysia [37], but is notably higher than previously reported rates within Egypt [38,39]. Crucially, the organism was frequently isolated from human samples including stool. As *A. baumannii* is not a primary enteropathogen, its isolation from stool and gastrointestinal samples in severely ill hospitalized patients most likely indicates heavy gastrointestinal (GI) tract colonization or carriage, rather than causative gastroenteritis. This heavy colonization represents a critical, silent reservoir for self-infection (leading to severe systemic infections elsewhere, such as pneumonia) and horizontal transmission within the hospital environment [35]. The highest isolation rates among human samples were found in throat swabs (66.7%), consistent with findings from Nepal [40]. These variations in prevalence across studies likely stem from differences in geographic location, climate, management systems, and specific sample types utilized [41,42,43].

The observed AMR profiles are alarming and reflect varied prescribing practices [44,45]. Our isolates displayed near-absolute resistance (100%) to ciprofloxacin and ceftriaxone, aligning with other regional reports [46,47]. Comparisons to global data show our strains possess higher resistance to key agents like gentamicin and sulfamethoxazole/trimethoprim but slightly lower resistance to imipenem [46]. The global rise of MDR strains has focused public health efforts on curbing the spread of the more severe XDR and PDR strains [48,49]. In our study, the investigated *A. baumannii* isolates were highly resistant, with 56.3% classified as PDR and 43.7% as XDR. While the high proportion of XDR is similar to findings in Iran (33.85%) [50] and India (27.32%) [51], the PDR rate (56.3%) is exceptionally high compared to reports from Egypt (2.2%) [52], Iran (3.93%) [53], and India (1.64%) [54]. Furthermore, all isolates exhibited MAR indices of 0.67 or greater, a finding substantially higher than observed in earlier Nigerian reports [55], underscoring the severity of resistance in this region. The elevated resistance observed, particularly to first-line agents like beta-lactams, aminoglycosides, and fluoroquinolones, is highly concerning as these are often the antibiotics of choice for treating human *A. baumannii* infections. Such high resistance creates a critical scarcity of viable treatment options. The likely driver of this extreme AMR burden in developing countries is the excessive and often unregulated use of antibiotics in both human medicine (as over-the-counter drugs) and animal husbandry (as growth promoters) [56,57,58,59]. This complex challenge is compounded by specific regional management and environmental factors in Egypt. In many peri-urban and rural Egyptian sectors, small-scale backyard poultry farming is deeply integrated into residential areas, generating high-density contact zones between humans, livestock, and synanthropic vectors [60]. Management practices such as the application of untreated poultry manure as agricultural fertilizer, agricultural runoff into shared water channels, and inadequate biosecurity barriers at farm perimeters facilitate the environmental persistence and dissemination of *A. baumannii*. Combined with potential gaps in clinical wastewater treatment systems, these environmental and anthropogenic pathways form an open, reciprocal loop where resistant clones continuously spill over between the clinic and the farm [61]. Therefore, urgent measures are needed to restrict antibiotic usage in both sectors. Additionally, the development and integration of substitute therapies, such as plant-derived medications, is desperately required to manage these highly resistant pathogens [62,63,64,65].

The cross-species dissemination of PDR *A. baumannii* across the clinical-poultry axis observed in the Sharqia Governorate (30.7° N, 31.63° E) mirrors an alarming global trajectory of high-risk clonal spread. While our study highlights the critical convergence of shared clones in North Africa, similar One Health challenges are echoing across diverse international landscapes. For instance, genomic surveillance in European settings like Poland, Germany, and France has increasingly documented the persistence of virulent, carbapenem-resistant *A. baumannii* lineages shifting between veterinary sectors and clinical environments [66,67,68]. Similarly, cross-species transmission pathways mapped in China, Canada, and the UK reinforce the reality that agricultural ecosystems act as significant reservoirs for clinical-grade resistance determinants [69,70].

This global paradigm is strongly supported by recent comparative genomic evidence from Vanishree et al. [71], who isolated and characterized multidrug-resistant A. baumannii from retail meat in India. Their work unveiled how food-producing animals and retail meat networks serve as vectors for high-risk international clones (such as IC-2 and IC-5) that harbor identical mobile resistance genes (bla_OXA-23_ and bla_NDM-1_) alongside robust virulence profiles. Our findings from Egypt align closely with this international trend, yet the transition from MDR to an absolute PDR status among 56.3% of our isolates indicates a severe regional escalation. Whether in European hospital wards, Canadian agricultural systems, or Egyptian poultry chains, *A. baumannii* is actively breaching species barriers. This global reality underscores that high-resolution, integrated One Health surveillance is no longer a localized luxury but an absolute international necessity [71].

The host’s immune system, relying on WBCs and neutrophils [50], suffered significant systemic impact in both hosts. Both chickens and humans presented with anemia, leukocytosis, neutrophilia, renal impairment, and highly elevated PCT and CRP levels, consistent with severe *A. baumannii* infection globally [50,51]. Both groups also showed clear signs of renal impairment (elevated urea and creatinine) and changes in protein metabolism, including low albumin and high globulin/total protein. This shift reflects an Acute Phase Response (APR), where the host’s liver ramps up production of acute phase proteins (largely globulins) while albumin synthesis is reduced [72]. This systemic protein imbalance is a direct marker of severe, ongoing inflammation.

We observed notable host-specific metabolic differences: hyperglycemia dominated in chickens (81.25%), whereas human patients displayed a mix of hypoglycemia (64.3%) and hyperglycemia (32.1%). Furthermore, cholesterol was elevated in most chickens (68.75%) but normal in all human patients. The severity of the pathological changes was directly linked to the resistance phenotype. Infections caused by Pandrug Resistant (PDR) isolates induced the most pronounced pathology [73], Specifically, these PDR strains showed a significant correlation with lower levels of Hb, PCV, RBCs, and albumin, and significantly higher levels of CRP, PCT, neutrophils, MCH, urea, and creatinine compared to less-resistant strains. This correlation is crucial: the higher total protein and globulin levels, coupled with lower albumin, were most pronounced in patients and birds infected with PDR strains. This finding underscores how the carriage of specific ARGs and the resulting extreme resistance phenotype drive a more profound and detrimental systemic inflammatory and metabolic response in the host. This association between extreme resistance and poor outcome aligns with human literature [74], and our work represents the first detailed report of such clinical impact in chickens.

In parallel with the severe systemic inflammation, we found that the expression levels in the serum of the proinflammatory cytokine (*IL-8*) and the tissue remodeling enzyme (*MMP-9*) were significantly upregulated in response to CRAB infection. *MMP-9* is crucial for neutrophil migration and matrix degradation, facilitating immune cell access to the site of infection [75,76]. However, its excessive activity also contributes to inflammation and tissue damage [75]. Mechanistically, *IL-8* acts as a potent chemoattractant that drives massive, uncontrolled neutrophilic infiltration into target organs [77]. While essential for initial pathogen containment, hyper-activation of the *IL-8* pathway triggers an oxidative burst and hyper-inflammatory cascade, directly echoing the elevated CRP and procalcitonin levels observed in our cohorts. This sustained neutrophilic presence stimulates the hyper-secretion of *MMP*-9, which degrades extracellular matrix components. In tissues such as the lungs, kidneys, and liver, excessive *MMP-9* activity disrupts endothelial-epithelial barriers, exacerbating capillary leakage, structural tissue destruction, and subsequent multi-organ dysfunction syndrome (MODS) [78]. This *MMP-9* upregulation, corroborated by similar findings in human pneumonia [79], suggests its major role in the lung’s response to *A. baumannii*. Similarly, the increased systemic expression of *IL-8* confirms its role in recruiting neutrophils [80], supporting the general activation of proinflammatory pathways (like those involving *IL-6* and *IL-1 beta*) observed in response to *A. baumannii* invasion [81]. Crucially, our work provides the first evidence of the impact of *A. baumannii* infection on the systemic expression levels of *MMP-9* and *IL-8* genes in infected chickens, demonstrating a conserved, highly destructive immunopathological pathway shared across divergent host species.

The utilization of last-resort antibiotics like carbapenems and colistin may be necessary because ESBL-producing Gram-negative bacteria are almost completely resistant to all β-lactams [43]. Following the initial identification of hospital-acquired *A. baumannii* isolates as an ESBL generator, community-associated infections have been identified globally [6]. Generally, it has been stated that *A. baumannii* carbapenem resistance is mostly linked to *bla_OXAs_* genes, with *bla_OXA-23_* being the most widely distributed one in most countries [82,83]. In this context, irrelevant to the source, all our tested 27 PDR CRAB isolates harbor *bla_OXAs_*, and *bla_OXA-23_* was the predominant gene (96.29%), followed by *bla_OXA-58_* (66.67%), *bla_OXA-48_* (51.85%), and *bla_OXA-24_* (48.15%). In Iran, among the tested clinical *A. baumannii* isolates, *bla_OXA-23_* was the most frequent gene (60%), followed by *bla_OXA-58_* and *bla_OXA-24_* (17.2% each) [84]. Likewise, a previous study conducted in Tunisia recorded that all clinical CRAB isolates carried *bla_OXA-23_* and *bla_OXA-51_*, while only 4% carried *bla_OXA-58_* [85]. In contrast, in Turkey, 100% [86] and 96% [87] of the tested clinical *A. baumannii* isolates harbored the *bla_OXA-23_* gene, followed by *bla_OXA-58_* (28.2 and 3%, respectively); however, none of the isolates possessed *bla_OXA-48_* and *bla_OXA-24_* genes [86,87]. Our 22 PDR CRAB isolates produced MBLs carbapenemases and possessed *bla_NDM_* (55.56%), *bla_KPC_* (48.15%), *bla_IMP_* (37.04%), and *bla_VIM_* (33.33%). Likewise, *bla_NDM_* was the most frequent gene (11.7%) among clinical CRAB isolates in Egypt, followed by *bla_KPC_* (10.7%) and *bla_VIM_* (0.5%), while *bla_IMP_* was not detected in any tested CRAB isolates [88]. Similarly, *bla_NDM_* was the prominent gene (23.36%) among clinical CRAB isolates in China, followed by *bla_VIM_* (1.64%) and *bla_IMP_* (0.82%), while *bla_KPC_* was not found in any tested CRAB isolates [89]. Nevertheless, a previous study in Thailand [90] was discordant with our findings, in which *bla_NDM_* was observed among 13.3% of tested clinical CRAB isolates, while *bla_KPC_*, *bla_VIM_*, and *bla_IMP_* were not found in any investigated CRAB isolates. Therefore, *bla_NDM_* was the dominant enzymatic mechanism of carbapenem resistance in hospitalized patients. In the present study, 22 PDR CRAB isolates (81.48%) harbored the *carO* gene, which is inconsistent with the result of a previous study conducted in Serbia (89.3%) [91]. Notably, all our CRAB isolates possessed *bla_OXAs_* genes in combination with MBL carbapenemase genes, which is inconsistent with the findings of a recent report conducted in Egypt, where all isolates possessed *bla_OXA51_* gene and 82.3% of the tested clinical CRAB isolates showed co-existence of more than one carbapenemase-encoding gene [92]; however, the prevalence of carbapenemase genes among PDR CRAB isolates from diseased chicken and their environment has not been investigated yet. Importantly, our reliance on target-specific PCR-based resistome screening did not encompass a structural investigation of mobile genetic elements (MGEs), such as plasmids, integrons, or insertion sequences (e.g., *ISAba1*, [93]). Consequently, the specific contribution of horizontal gene transfer (HGT) relative to vertical clonal dissemination in shaping these multi-carbapenemase profiles cannot be completely decoupled in this study, representing an administrative limitation that warrants caution in mechanistic interpretations.

This work presents the first report on the possible correlation of PDR *A. baumannii* isolates from diseased chickens, their environment, and hospitalized patients with ERIC-PCR and MLST. ERIC-PCR fingerprinting as an initial genotyping method revealed significant similarities between *A. baumannii* isolates from diseased chickens and those from hospitalized patients. This finding is consistent with a recent Egyptian study that also demonstrated significant genetic relatedness among *A. baumannii* isolates from poultry and hospitalized patients by ERIC-PCR [94]. While another Iranian study using ERIC-PCR showed greater diversity among human *A. baumannii* isolates, suggesting multiple subtypes contribute to infection [95], our results point towards a possible link between animal and human isolates. Herein, we conducted MLST on five PDR chicken and human *A. baumannii* isolates sharing the same ET (ET19 and ET20) to confirm the potential transmission between chicken and humans and to elucidate the direction and mechanisms of this transmission. MLST analysis confirmed ERIC-PCR findings, where two and three isolates from human and chicken sources belonged to ST1410 and ST1828, respectively, which confirmed the potential cross-species link between human and chicken isolates. Accordingly, a recent report revealed that MDR *A. baumannii* ST25 has been detected in both animals and humans, proposing cross-contamination among the two origins [96]. Likewise, a previous German study reported ST1410 among one *A. baumannii* isolate from animal origin [97].

Despite these significant phylogenetic alignments, we must acknowledge the inherent limitations of our molecular typing framework. While ERIC-PCR provides rapid, highly discriminatory epidemiological sorting for local outbreaks, it can suffer from inter-laboratory reproducibility constraints and fails to clarify long-term evolutionary trends. Conversely, while MLST successfully established high-confidence cross-species sequence clusters (ST1410 and ST1828), it tracks changes across seven highly conserved housekeeping genes, which potentially masks vital micro-evolutionary micro-diversities or shifts within the accessory genome. Furthermore, because MLST was selectively restricted to a representative subset of five isolates from matching cross-species ERIC clusters rather than deployed across the entire 48-isolate repository, our conclusions regarding the exact rate and definitive direction of cross-species transmission remain preliminary.

## 5. Conclusions

This study establishes crucial baseline data on the alarming prevalence and complex resistance mechanisms of XDR and PDR *A. baumannii* (CRAB) across human, poultry, and environmental sources. The core finding is the molecular linkage confirmed by MLST analysis (supporting initial ERIC-PCR results), revealing shared genotypes between human and chicken PDR CRAB isolates, which strongly indicates a potential cross-species transmission pathway. These linked strains exhibit extremely complex resistance, highlighted by the frequent co-occurrence of multiple carbapenemase genes (OXA-type, MBL, and *bla_KPC_*). Significantly, these shared PDR strains were associated with severe systemic pathology in both hosts, demonstrated by distinct hematological and biochemical changes (including anemia and renal dysfunction) and the upregulation of inflammatory markers *(IL-8* and *MMP-9*). Given the high prevalence of transmissible PDR *A. baumannii* and their observed clinical impact, an immediate and comprehensive One Health strategy is imperative in Egypt. This response must include coordinated CRAB surveillance and control across human and poultry populations, stringent infection control, and judicious antibiotic stewardship to mitigate this escalating public health threat.

To fully overcome the limitations of traditional locus-based epidemiological typing and PCR profiling, future surveillance architectures must incorporate high-resolution Whole-Genome Sequencing (WGS). Utilizing WGS in subsequent cohorts will allow for precise core-genome single-nucleotide polymorphism (SNP) typing, definitive localization of multi-drug resistance cassettes within specific plasmid backbones, and mapping of critical insertion sequences. This advanced genomic approach will expand the interpretive scope of future surveillance, allowing researchers to accurately map the evolutionary networks of these highly critical lineages at the animal–human-environment interface.

## Figures and Tables

**Table 1:** Characteristics of diseased chickens, their environment, and hospitalized patients’ samples in the current study.

Variables	No. (Prevalence, %)
Chickens (*n*= 43)	
**Clinical condition**	
Intestinal/Diarrheal Symptoms	31 (72.1)
Respiratory manifestations	3 (6.9)
Severe respiratory manifestations	3 (6.9)
Severe respiratory manifestations with decreased egg production	6 (13.9)
**Age group (8–12 Weeks)**	
<9 Weeks	19 (44.2)
≥9 Weeks	24 (55.8)
**Breed**	
Baladi	17 (39.5)
Broiler	17 (39.5)
Layer	9 (20.9)
**Sample type**	
Cloacal swabs	22 51.2)
Fecal samples	9 (20.9)
Nasal swabs	12 (48.8)
Chicken environment (*n* = 24)	
**Sample type**	
Chicken house	10 (41.7)
Equipment	4 (16.7)
Food	5 (20.8)
Water	5 (20.8)
Humans (*n* = 78)	
**Clinical condition**	
Bloodstream infection	20 (25.6)
UTI-like Symptoms/Bacteriuria	31 (39.7)
Gastrointestinal symptoms	13 (16.7)
Severe gastrointestinal symptoms	2 (2.6)
Pyogenic wound infection	3 (3.8)
Respiratory manifestations	2 (2.6)
Severe respiratory manifestations (VAP)	7 (8.9)
**Age group (28–62 Years)**	
<45 Years	39 (50)
≥45 Years	39 (50)
**Gender**	
Male	34 (43.6)
Female	44 (56.4)
**Sample type**	
Blood	24 (30.8)
Nasopharyngeal swabs	4 (5.1)
Sputum	3 (3.8)
Stool	7 (8.9)
Throat swabs	6 (7.7)
Urine	31 (16.7)
Wound swabs	3 (3.8)

VAP: ventilator-associated pneumonia.

**Table 2:** Analysis of hematological and biochemical parameters among diseased chickens and humans infected with *Acinetobacter baumannii*.

Parameter	Chicken (*n* = 16)	Human (*n* = 28)	*p*-Value	Total (*n* = 44)	Normal Range of Investigated Parameters	
					Chicken	Human
Blood glucose level, mg/dL	285.38 ± 18.18	118.32 ± 20.3	<0.0001 ***	179.07 ± 18.9	150–200	Fasting: 70–99, 2 h postprandial blood sugar: <140
CRP, mg/dL	23.31 ± 1.46	47.21 ± 2.62	<0.0001 ***	38.52 ± 2.47	<1	<0.5 (normal), 0.5–1.0 (low-grade inflammation), >1.0 (high-grade inflammation)
PCT, ng/mL	1.32 ± 0.05	1.36 ± 0.01	0.467	1.35 ± 0.019	<0.5	<0.5 (normal), >0.5 (bacterial infection)
CBC						
WBCs, 10^3^ cells/mm^3^ blood	38.11 ± 0.86	18.7 ± 0.62	<0.0001 ***	25.76 ± 1.51	15–30	4.5–11
Neutrophils, %	92.54 ± 2.07	96.72 ± 3.6	0.321	95.20 ± 2.41	25–60	40–60
RBCs, 10^6^ cells/mm^3^ blood	2.19 ± 0.09	2.66 ± 0.07	<0.0001 ***	2.49 ± 0.07	3–4.5	4–5.4 for women, 4.5–5.9 for men
PCV, %	24.48 ± 0.53	28.39 ± 0.52	<0.0001 ***	27.03 ± 0.48	35–45	36–48% for women, 40–54% for men
Hb, g/dL	8.87 ± 0.24	9.29 ± 0.15	0.126	9.14 ± 0.13	12–16	12–16 for women, 13.5–17.5 for men
MCH, pg/cell	40.81 ± 0.88	35.32 ± 0.5	<0.0001 ***	37.31 ± 0.60	25–35	27–31
Kidney function test						
Creatinine, mg/dL	2.88 ± 0.17	2.83 ± 0.13	0.795	2.85 ± 0.10	0.5–1.5	0.7–1.3 for men, and 0.6–1.1 for women
Urea, mg/dL	40.89 ± 2.09	35.46 ± 1.1	0.032 *	37.44 ± 1.10	15–30	10–20
Liver function test						
Albumin, g/L	20.13 ± 0.63	23.36 ± 1.1	0.018 *	22.19 ± 0.79	30–50	35–50
Globulin, g/L	63.29 ± 4.2	66.45 ± 2.6	0.512	65.32 ± 0.29	20–40	23–35
A/G	0.35 ± 0.03	0.38 ± 0.03	0.494	0.37	>1	1.5–2.5
Total protein, g/L	83.42 ± 3.75	89.81 ± 1.8	0.139	87.49 ± 1.82	60–80	60–83
Cholesterol, mg/dL	270.8 ± 12.3	79.37 ± 6.2	<0.0001 ***	148.98 ± 15.24	150–250	<200

CBC: complete blood count; WBCs: white blood cells; RBCs: red blood cells; PCV: packed cell volume; Hb: hemoglobin; MCH: mean corpuscular hemoglobin; CRP: C-reactive protein; PCT: procalcitonin; A/G: albumin/globulin ratio. * *p* < 0.05, *** *p* < 0.001.

**Table 3:** Multilocus sequence typing (MLST) among chicken and human PDR *Acinetobacter baumannii* isolates.

Sequence Type (ST)	Isolate Code No.	Source	Allelic Profile	Carbapenemase- and ESBL-Encoding Genes
1410	132Hu	Human	40-3-2-1-2-2-2	*bla_KPC_*, *bla_NDM_*, *bla_OXA-23_*, *bla_OXA-48_*, *bla_OXA-24_*, *carO*
1410	104Ch	Chicken	40-3-2-1-2-2-2	*bla_IMP_*, *bla_NDM_*, *bla_OXA-23_*, *bla_OXA-48_*, *bla_OXA-58_*, *carO*
1828	90Hu	Human	3-2-2-2-7-2-2	*bla_OXA-23_*, *bla_OXA-48_*, *bla_OXA-58_*, *carO*
1828	63Ch	Chicken	3-2-2-2-7-2-2	*bla_NDM_*, *bla_OXA-23_*, *bla_OXA-48_*, *bla_OXA-24_*, *carO*
1828	64Ch	Chicken	3-2-2-2-7-2-2	*bla_IMP_*, *bla_VIM_*, *bla_OXA-23_*, *bla_OXA-24_*, *carO*

## Data Availability

The datasets supporting the findings of this study are available within the article and its Supplementary Materials. Nucleotide sequences have been assigned GenBank accession numbers PZ241909–PZ241943. Correspondence and requests for further materials should be addressed to A.S.E-D.

## Supplementary Materials

The following supporting information can be downloaded at https://www.mdpi.com/article/10.3390/microorganisms14071409/s1. Supplementary Table S1. Oligonucleotide primer sequences utilized in molecular assays [12,13, 23,26,98,99,100,101,102], Supplementary Table S2. Prevalence of *A. baumannii* from various sample sources, types, and clinical conditions; Supplementary Table S3. Univariate logistic regression model for each sample source; Supplementary Table S4. Antimicrobial resistance patterns of *Acinetobacter baumannii* isolated from various sources via the broth microdilution method; Supplementary Table S5. Antimicrobial resistance patterns of *Acinetobacter baumannii* isolated from various sources; Supplementary Table S6. Minimal inhibitory concentrations of tested antimicrobials against *Acinetobacter baumannii* isolates from different sources; Supplementary Table S7. Multiple antibiotic resistance indices (MAR) of *Acinetobacter baumannii* isolates from various sample sources; Supplementary Figure S1. Prevalence of *Acinetobacter baumannii* from diseased chickens, their environment, and hospitalized patients in the study area; Supplementary Figure S2. Stacked bar graph displaying the prevalence of *Acinetobacter baumannii* from various sample types (A), and clinical conditions (B) of diseased chickens, and hospitalized patients in the study area; Supplementary Figure S3. Multiple antibiotic resistance indices (MAR) (A), the distribution of resistant antimicrobial agents (B), and classes (C) among *Acinetobacter baumannii* isolates from various sources in the study area; Supplementary Figure S4. Analysis of hematological (A), and biochemical (B) parameters of diseased chicken, and humans infected with *Acinetobacter baumannii* isolates belonging to various resistance categories; Supplementary Figure S5. Analysis of hematological and biochemical parameters among diseased chickens, and humans infected with *Acinetobacter baumannii;* Supplementary Figure S6. Hierarchical clustering dendrogram revealing the binary distances among the investigated variables (A), and PDR *Acinetobacter baumannii* isolates (B) based on the frequency distribution of carbapenemase genes, hematological, and biochemical parameters, and the expression of *IL-8*, and *MMP-9* genes, Supplementary Figure S7. PCA (Principal component analysis) biplot revealing the overall distribution of PDR *Acinetobacter baumannii* isolates from diseased chickens and hospitalized patients based on the frequency distribution of carbapenemase genes, hematological, and biochemical parameters, and the expression of *IL-8* and *MMP-9* genes.
