# Supplementary material for: Cross-Species Dissemination of Pandrug-Resistant Acinetobacter baumannii in Humans and Poultry in Egypt: Unveiling Shared Clones, Resistance Mechanisms, and Severe Clinical Outcomes
Source: Microorganisms. 2026 Jun 26;14(7):1409. doi: 10.3390/microorganisms14071409 (PMC13414185; doi:10.3390/microorganisms14071409)
Supplement: Supplementary file 1 [file microorganisms-14-01409-s001.zip › microorganisms-4364501-supplementary.pdf]

Supplementary Material

Supplementary Table S1. Oligonucleotide primer sequences utilized in molecular assays

Target gene	Primers sequences (5′ → 3′)	Amplified segment (bp)	Annealing (°C)	Reference
Acinetobacter baumannii				
16S rRNA	F: CAGCTCGTGTCGTGAGATGT	150	55	[12]
	R: CGTAAGGGCCATGATGACTT			
rpoB	F: ACGCCTAAAGGTGAAACTCAGTTAA	105	60	[13]
	R: GTACCAGATGGAACACGTAAAGATG			
rpoD	F: GTTGCTGAAGAAGAAGCTGCTG	105	60	[13]
	R: ACTGTACCCATTTCACGCATGTA			
fabD	F: TTGCAGAAGCTTTGGAACAAACT	115	60	[13]
	R: CGTAATTGAGCAACATCGGTAGC			
**Inflammation-related genes (Chicken)**				
β-actin	F: TTCTTTTGGCGCTTGACTCA	88	60	[98]
	R: GCGTTCGCTCCAACATGTT			
MMP-9	F: ATGCCTGCATGGAGAAGAAC	106	60	[98]
	R: CCTGATTTCCAGAAGGACGA			
IL-8	F: CCGATGCCAGTGCATAGAGA	199	60	[99]
	R: GGTGTCTGCCTTGTCCAGAA			
**Inflammation-related genes (Human)**				
β-actin	F: ATGGAGTCCTGTGGCATCC	155	60	This study

	R: AGGGCAGTGATCTCCTTCTG			(GenBank: M28424.1)
MMP-9	F: TCTTCCCTGGAGACCTGAGA	175	60	This study
	R: CTATCCAGCTCACCGGTCTC			(GenBank: NM_004994.3)
IL-8	F: TTCAGAGACAGCAGAGCACA	150	60	This study
	R: TACCTTCACACAGAGCTGCA			(GenBank: MN930489.1)
**Carbapenem resistance genes**				
$bla_{OXA\text{-}23}$	F: ATCGGATTGGAGAACCAGA	501	53	[100]
	R: ATTTCTGACCGCATTTCCAT			
$bla_{OXA\text{-}58}$	F: AAGTATTGGGGCTTGTGCTG	599	53	[100]
	R: CCCCTCTGCGCTCTACATAC			
$bla_{OXA\text{-}24}$	F: GGTTAGTTGGCCCCCTTAAA	246	53	[100]
	R: AGTTGAGCGAAAAGGGGATT			
bla_{VIM}	F: CCGTAGAACAAGCAGGCATC	230	60	This study
	R: GCTGCCCTACCTCTCACTAG			(GenBank: PP458162.1)
$bla_{OXA\text{-}48}$	F: GCGTGGTTAAGGATGAACAC	438	60	[101]
	R: CATCAAGTTCAACCCAACCG			
bla_{KPC}	F: CGTCTAGTTCTGCTGTCTTG	798	60	[101]
	R: CTTGTCATCCTTGTTAGGCG			
bla_{NDM}	F: GGTTTGGCGATCTGGTTTTC	621	60	[101]
	R: CGGAATGGCTCATCACGATC			
bla_{IMP}	F: GGAATAGAGTGGCTTAAYTCTC	232	50	[101]
	R: GGTTTAAYAAAACAACCACC			

carO	F: ATTGTAGAAAGCTGAGACAT	Variable	50	[102]
	R: ATTTCTYTATGCTCACCTGA			
**ERIC-PCR**				
ERIC	F: ATGTAAGCTCCTGGGGATTCAC	Variable		[23]
	R: AAGAAGTGACTGGGGTGAGCG			
**MLST**				
cpn60	F: ACTGTACTTGCTCAAGC	405	50	[26]
	R: TTCAGCGATGATAAGAAGTGG			
fusA	F: ATCGGTATTTCTGCKCACATYGAT	633	50	[26]
	R: CCAACATACKYTGWACACCTTTGTT			
gltA	F: AATTTACAGTGGCACATTAGGTCCC	483	50	[26]
	R: GCAGAGATACCAGCAGAGATACACG			
pyrG	F: GGTGTTGTTTCATCACTAGGWAAAGG	297	50	[26]
	R: ATAAATGGTAAAGAYTCGATRTCACCMA			
recA	F: CCTGAATCTTCYGGTAAAAC	372	50	[26]
	R: GTTTCTGGGCTGCCAAACATTAC			
rplB	F: GTAGAGCGTATTGAATACGATCCTAACC	330	50	[26]
	R: CACCACCACCRTGYGGGTGATC			
rpoB	F: GGCGAAATGGC(AGT)GA(AG)AACCA	256	50	[26]
	R: GAAGTCCTTCGAAGTTGTAACC			

Supplementary Table S2. Prevalence of *A. baumannii* from various sample sources, types, and clinical conditions

Sample source (No.)	No of positive samples (%)[a]	*p*-value
**Chickens (43)**	16 (37.2)	
**Clinical condition**		
Intestinal/Diarrheal Symptoms (31)	15 (48.4)	0.103
Respiratory manifestations (3)	0	
Severe respiratory manifestations (3)	0	
Severe respiratory manifestations with decreased egg production (6)	1 (16.7)	
**Age group (8-12 Weeks)**		
<9 Weeks (19)	6 (31.6)	0.36
≥ 9 Weeks (24)	10 (41.7)	
**Breed**		
Baladi (17)	4 (23.5)	0.359
Broiler (17)	8 (47.1)	
Layer (9)	4 (44.4)	
**Sample type**		
Cloacal swabs (22)	10 (45.5)	0.054
Fecal samples (9)	5 (55.6)	
Nasal swabs (12)	1 (8.3)	
**Chicken environment (24)**	4 (16.7)	
**Sample type**		
Chicken house (10)	0	0.218
Equipment (4)	1 (25)	
Food (5)	2 (40)	
Water (5)	1 (20)	
**Humans (78)**	28 (35.9)	
**Clinical condition**		
Bloodstream infection (20)	7 (35)	0.407
UTI-like Symptoms/Bacteriuria (31)	11 (35.5)	
Gastrointestinal symptoms (13)	2 (15.4)	
Severe gastrointestinal symptoms (2)	2 (100)	
Pyogenic wound infection (3)	1 (33.3)	
Respiratory manifestations (2)	0	
Severe respiratory manifestations (VAP) (7)	5 (71.4)	
**Age group (28-62 Years)**		
<45 Year (39)	13 (33.3)	0.47
≥45 Year (39)	15 (38.5)	
**Gender**		
Male (34)	14 (41.2)	0.268
Female (44)	14 (31.8)	
**Sample type**		
Blood (24)	7 (29.2)	0.379
Nasopharyngeal swabs (4)	0	

Sputum (3)	1 (33.3)
Stool (7)	4 (57.1)
Throat swabs (6)	4 (66.7)
Urine (31)	11 (35.5)
Wound swabs (3)	1 (33.3)

[a] The isolation rates were calculated regarding the total number of the investigated samples. VAP: ventilator-associated pneumonia.

Supplementary Table S3. Univariate logistic regression model for each sample source

	β-coefficient	Odds ratio	*p*-value
**Chickens**			
**Breed**			
(Intercept)	-1.179	0.308	0.039*
Baladi	0	1	0.334
Broiler	1.061	2.889	0.157
Layer	0.956	2.6	0.278
**Sample type**			
(Intercept)	-0.182	0.833	0.67
Cloacal swabs	0	1	0.095
Fecal samples	0.405	1.5	0.61
Nasal swabs	-2.216	0.109	0.05
**Age**			
(Intercept)	-1.964	0.418	0.14
Age	0.159	0.548	1.172
**Clinical condition**			
(Intercept)	-0.065	0.938	0.857
Intestinal/Diarrheal Symptoms	0	1	0.616
Respiratory manifestations	-21.138	0	0.999
Severe respiratory manifestations	-21.138	0	0.999
Severe respiratory manifestations with decreased egg production	-1.545	0.213	0.18
**Humans**			
**Age**			
(Intercept)	-0.499	0.607	0.654
Age	-0.002	0.998	0.941
**Gender**			
(Intercept)	-0.762	0.467	0.019*
Female	0	1	1
Male	0.405	1.5	0.394
**Clinical condition**			
(Intercept)	-0.619	0.538	0.187
Bloodstream infection	0	1	0.499
UTI-like Symptoms/Bacteriuria	0.021	1.021	0.972
Gastrointestinal symptoms	-1.086	0.338	0.228
Pyogenic wound infection	-0.074	0.929	0.955
Respiratory manifestations	-20.584	000	0.999
Severe gastrointestinal symptoms	21.822	3000167565.3	0.999
Severe respiratory manifestations (VAP)	1.535	4.643	0.109
**Sample Type**			
(Intercept)	-0.887	0.412	0.048*
Blood	0	1	0.701
Nasopharyngeal swabs	-20.316	0	0.999
Sputum	0.194	1.214	0.882
Stool	1.175	3.238	0.185

Throat swabs	1.580	4.857	0.105
Urine	0.289	1.336	0.621
Wound swabs	0.194	1.214	0.882
**Chicken environment**			
**Sample type**			
(Intercept)	-21.203	0	0.999
Chicken house	0	1	0.916
Equipment	20.104	538491617.639	0.999
Food	20.797	1076983235.279	0.999
Water	19.817	403868713.230	0.999

* Significant. VAP: ventilator-associated pneumonia.

Supplementary Table S4. Antimicrobial resistance patterns of *Acinetobacter baumannii* isolated from various sources via the broth microdilution method

AMC	AMA	No. of resistant *Acinetobacter* isolates (%)[a]			*p*-value	Total no. of resistant *Acinetobacter* isolates (n=48)
		Chicken (n=16)	Chicken environment (n=4)	Human (n=28)		
β-lactam combination agents	TZP	15 (93.75)	4 (100)	28 (100)	0.417	47 (97.92)
Cephalosporin IV	FEP	15 (93.75)	4 (100)	28 (100)	0.417	47 (97.92)
Cephamycin	FOX	16 (100)	4 (100)	27 (96.43)	1	47 (97.92)
Carbapenem	IMP	13 (81.25)	4 (100)	28 (100)	0.048 *	45 (93.75)
	MEM	14 (87.5)	4 (100)	27 (96.43)	0.65	45 (93.75)
Aminoglycosides	CN	16 (100)	4 (100)	28 (100)	NA	48 (100)
	AK	11 (68.75)	4 (100)	20 (71.43)	0.566	35 (72.92)
	TOB	15 (93.75)	4 (100)	27 (96.43)	1	46 (95.83)
Fluoroquinolone	CIP	16 (100)	4 (100)	28 (100)	NA	48 (100)
	LEV	16 (100)	4 (100)	27 (96.43)	1	47 (97.92)

Folate pathway antagonists	SXT	16 (100)	4 (100)	28 (100)	NA	48 (100)
Polymyxins	CT	16 (100)	4 (100)	28 (100)	NA	48 (100)
Tetracyclines	TE	15 (93.75)	4 (100)	28 (100)	0.417	47 (97.92)
	MH	16 (100)	4 (100)	28 (100)	NA	48 (100)
Glycylcyclines	TGC	13 (81.25)	4 (100)	22 (78.57)	0.672	39 (81.25)

[a] The isolation rates were calculated regarding the total number of the investigated isolates. AMC: antimicrobial class, AMA: antimicrobial agent, TZP: piperacillin/tazobactam, FOX: cefoxitin, FEP: cefepime, IMP: imipenem, MEM: meropenem, CN: gentamicin, AK: amikacin, TOB: tobramycin, CIP: ciprofloxacin, LEV: levofloxacin, SXT: sulfamethoxazole/trimethoprim, CT: colistin, TGC: tigecycline, TE: tetracycline, MH: minocycline, NA: non-applicable, * $p < 0.05$.

Supplementary Table S5. Antimicrobial resistance patterns of *Acinetobacter baumannii* isolated from various sources

AMC	AMA	No. of resistant *Acinetobacter* isolates (%)[a]			*p*-value	Total no. of resistant *Acinetobacter* isolates (n=48)
		Chicken (n=16)	Chicken environment (n=4)	Human (n=28)		
Penicillins	P	16 (100)	4 (100)	28 (100)	NA	48 (100)
	AM	15 (93.75)	4 (100)	23 (82.14)	0.415	42 (87.5)
β-lactam combination agents	TZP	15 (93.75)	4 (100)	28 (100)	0.417	47 (97.92)
Cephalosporin I	KZ	16 (100)	4 (100)	27 (96.43)	1	47 (97.92)
Cephalosporin III	CTX	13 (81.25)	4 (100)	28 (100)	0.048*	45 (93.75)
	CRO	16 (100)	4 (100)	28 (100)	NA	48 (100)
	CAZ	15 (93.75)	4 (100)	28 (100)	0.417	47 (97.92)
Cephalosporin IV	FEP	15 (93.75)	4 (100)	28 (100)	0.417	47 (97.92)
Cephamycin	FOX	16 (100)	4 (100)	27 (96.43)	1	47 (97.92)
Carbapenem	IMP	13 (81.25)	4 (100)	28 (100)	0.048*	45 (93.75)
	MEM	14 (87.5)	4 (100)	27 (96.43)	0.65	45 (93.75)
Aminoglycosides	CN	16 (100)	4 (100)	28 (100)	NA	48 (100)
	AK	11 (68.75)	4 (100)	20 (71.43)	0.566	35 (72.92)
	TOB	15 (93.75)	4 (100)	27 (96.43)	1	46 (95.83)
Fluoroquinolone	CIP	16 (100)	4 (100)	28 (100)	NA	48 (100)
	LEV	16 (100)	4 (100)	26 (92.86)	0.603	46 (95.83)
Folate pathway antagonists	SXT	16 (100)	4 (100)	28 (100)	NA	48 (100)
Polymyxins	CT	15 (93.75)	4 (100)	28 (100)	0.417	47 (97.92)
Tetracyclines	TE	15 (93.75)	4 (100)	28 (100)	0.417	47 (97.92)
	DO	14 (87.5)	4 (100)	26 (92.86)	0.731	44 (91.67)
	MH	16 (100)	4 (100)	28 (100)	NA	48 (100)
Glycylcyclines	TGC	13 (81.25)	4 (100)	22 (78.57)	0.132	39 (81.25)

[a] The isolation rates were calculated regarding the total number of the investigated isolates.
AMC: antimicrobial class, AMA: antimicrobial agent, P: penicillin, AM: ampicillin, TZP: piperacillin/tazobactam, CTX: cefotaxime, CRO: ceftriaxone, FOX: cefoxitin, KZ: cefazolin, CAZ: ceftazidime, FEP: cefepime, IMP: imipenem, MEM: meropenem, CN: gentamicin, AK: amikacin, TOB: tobramycin, CIP: ciprofloxacin, LEV: levofloxacin, SXT: sulfamethoxazole/trimethoprim, CT: colistin, TGC: tigecycline, TE: tetracycline, DO: doxycycline, MH: minocycline, NA: non-applicable, * $p< 0.05$.

Supplementary Table S6. Minimal inhibitory concentrations of tested antimicrobials against *Acinetobacter baumannii* isolates from different sources

AMA	No. of *A. baumannii* isolates showing MIC values of the tested antimicrobials (µg/mL)													MIC_{50}	MIC_{90}
	0.25	0.5	1	2	4	8	16	32	64	128	256	512	1024		
TZP			1								13	28	6	512	1024
FEP			1									2	45	1024	1024
FOX				1							10	17	20	512	1024
IMP		1	1	1						16	5	9	15	512	1024
MEM		1					2			1			44	1024	1024
CN										2		9	37	1024	1024
AK		1	4	5	2						6	12	18	1024	1024
TOB							2		1	9	9	8	19	512	1024
CIP													48	1024	1004
LEV							1		1	3	3	17	23	512	1024
SXT													48	1024	1024
CT									1	11	26	7	3	256	512
TE				1									47	1024	1024
MH													48	1024	1024
TGC							9		2	9	23	2	3	256	512

AMA: antimicrobial agent, TZP: piperacillin/tazobactam, CRO: ceftriaxone, FOX: cefoxitin, FEP: cefepime, IMP: imipenem, MEM: meropenem, CN: gentamicin, AK: amikacin, TOB: tobramycin, CIP: ciprofloxacin, LEV: levofloxacin, SXT: sulfamethoxazole/trimethoprim,

CT: colistin, TGC: tigecycline, TE: tetracycline, MH: minocycline, MIC: minimum inhibitory concentration, MIC_{50} = (n x 0.5), MIC_{90} = (n x 0.9).

Supplementary Table S7. Multiple antibiotic resistance indices (MAR) of *Acinetobacter baumannii* isolates from various sample sources

MAR index	No. of resistant AMA	No. of resistant AMC	No. of resistant *Acinetobacter* isolates (%)			*p*-value	Total no. of resistant *Acinetobacter* isolates (n=48)	Resistance category
			Chicken (n= 16)	Chicken environment (n= 4)	Human (n=28)			
0.67	10	9	1 (6.25)	-	-	0.417	1 (2.08)	XDR
0.73	11	9	1 (6.25)	-	-	0.417	1 (2.08)	XDR
		10	1 (6.25)	-	-	0.417	1 (2.08)	XDR
0.8	12	9	-	-	1 (3.57)	0.417	1 (2.08)	XDR
0.87	13	9	1 (6.25)	-	1 (3.57)		2 (4.16)	XDR
0.93	14	9	-	-	7 (25)	0.046*	7 (14.58)	XDR
		10	2 (12.5)	-	6 (21.43)	0.608	8 (16.67)	XDR
1	15	11	10 (62.5)	4 (100)	13 (46.43)	0.102	27 (56.25)	PDR

AMC: antimicrobial class, AMA: antimicrobial agent, MAR: multiple antibiotic resistance, MDR: multidrug-resistant, XDR: extensively drug-resistant, PDR: pandrug-resistant. **p*<0.05.

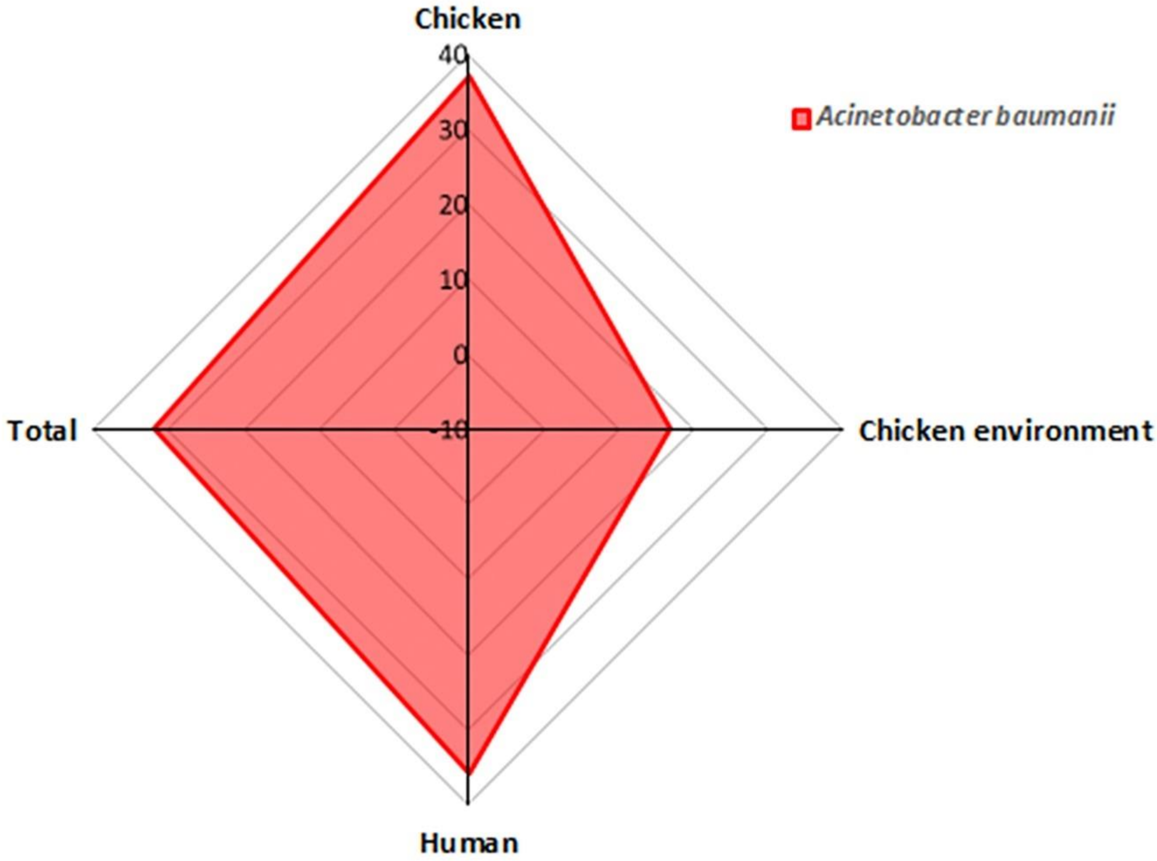

Supplementary Figure S1. Prevalence of *Acinetobacter baumannii* from diseased chickens, their environment, and hospitalized patients in the study area.
[a] The isolation rates were calculated regarding the total number of the investigated samples.

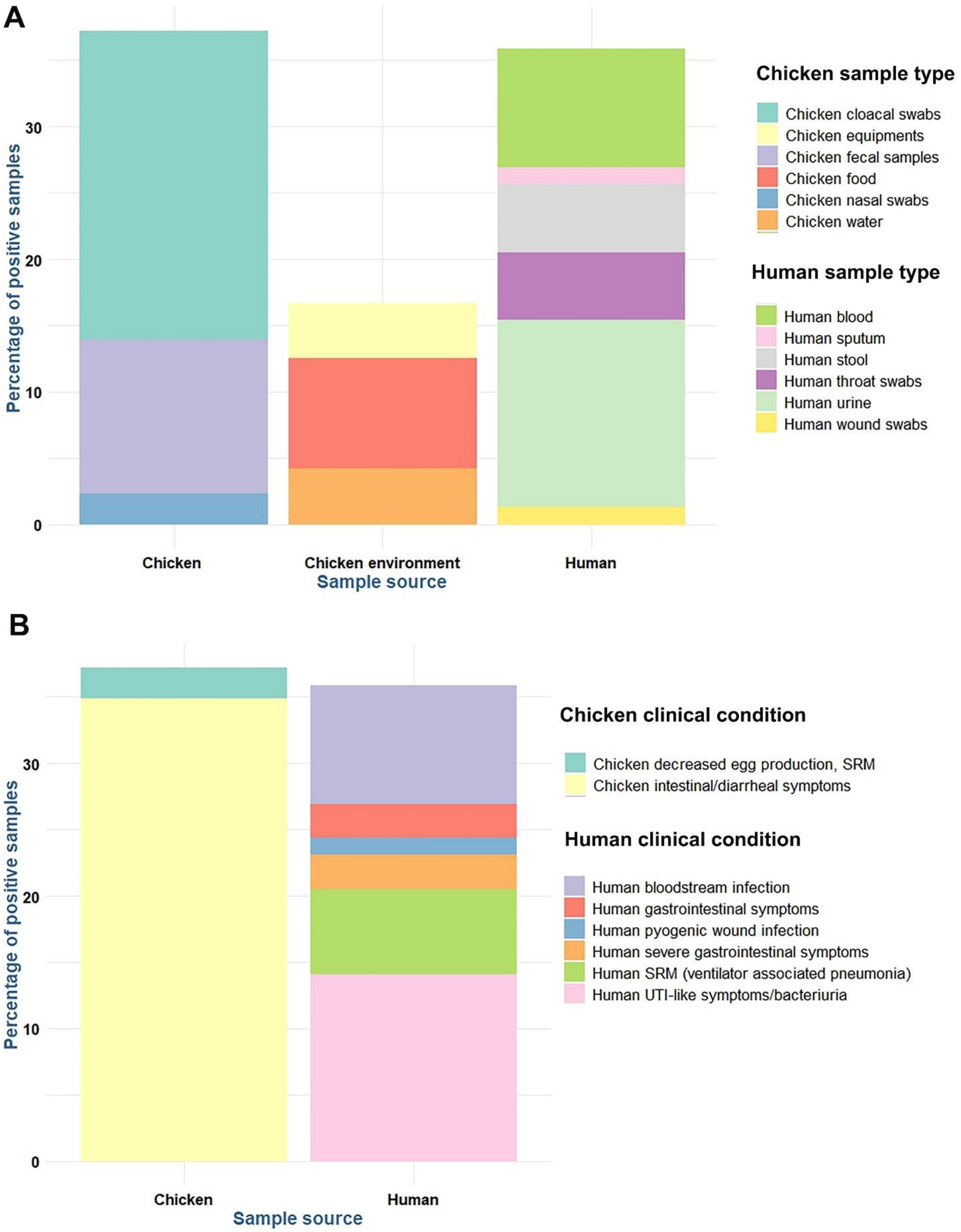

Supplementary Figure 2. Stacked bar graph displaying the prevalence of *Acinetobacter baumannii* from various sample types (A), and clinical conditions (B) of diseased chickens, and hospitalized patients in the study area. * The isolation rates of various sample sources

were calculated regarding the total number of the investigated samples, and subcolumns are calculated as part of the total column. SRM: severe respiratory manifestations.

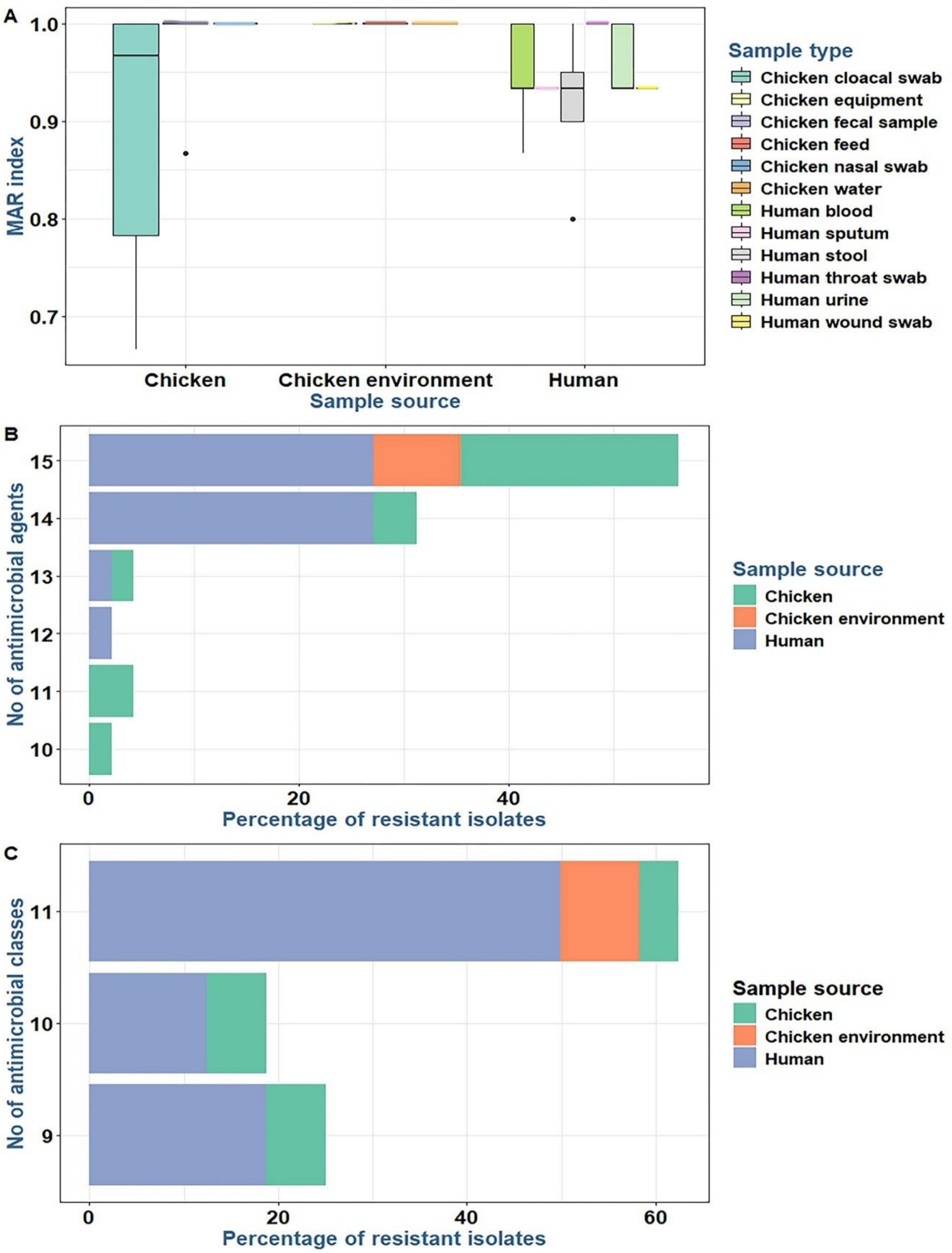

Supplementary Figure S3. Multiple antibiotic resistance indices (MAR) (**A**), the distribution of resistant antimicrobial agents (**B**), and classes (**C**) among *Acinetobacter baumannii* isolates from various sources in the study area. * In the stacked bars, the

frequency was calculated concerning the total number of the examined isolates (n= 48) for each parameter, and subcolumns are calculated as a part of the total column.

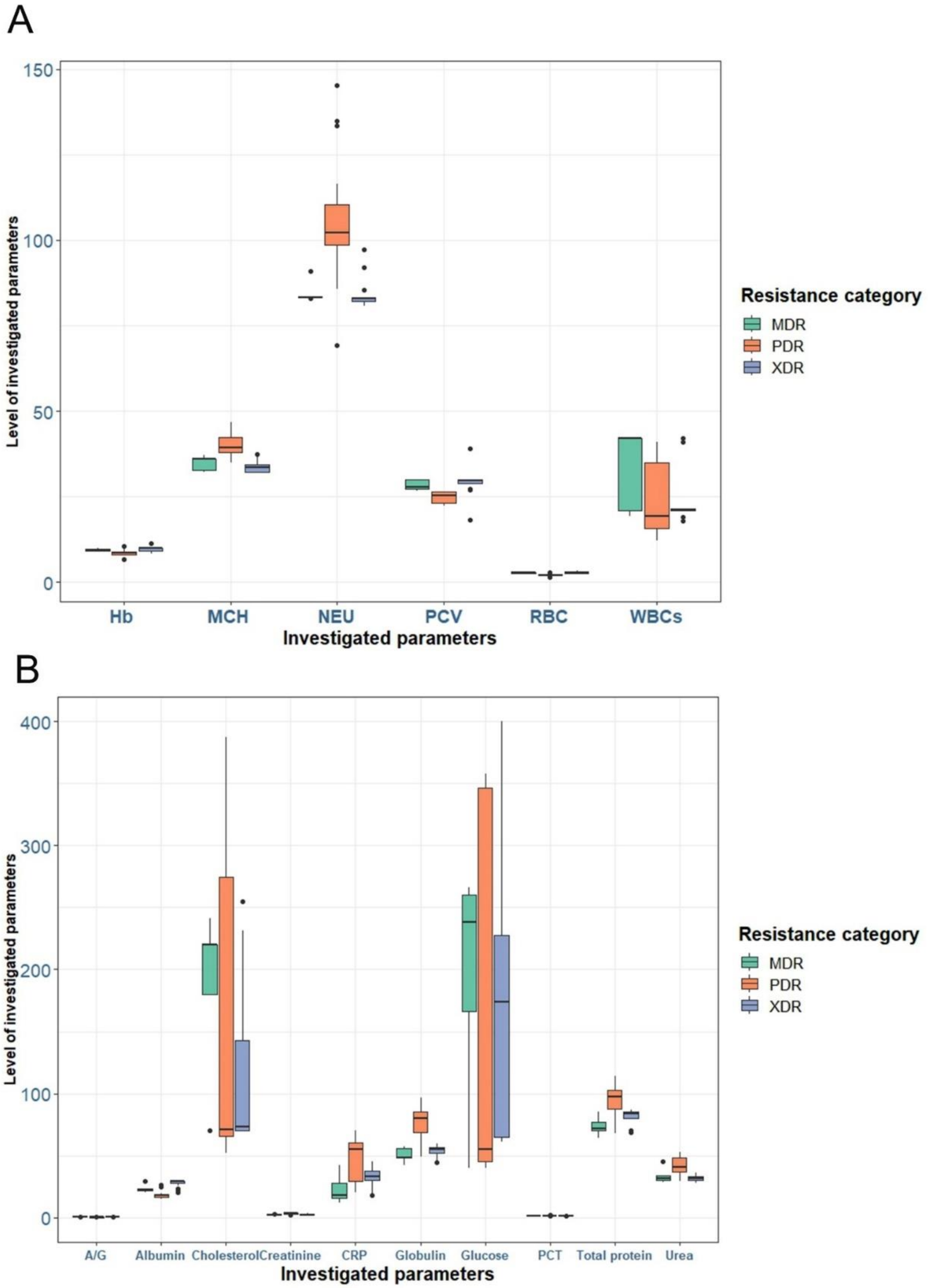

Supplementary Figure S4. Analysis of hematological (A), and biochemical (B) parameters of diseased chicken, and humans infected with *Acinetobacter baumannii* isolates belonging to various resistance categories. WBCs: white blood cells, RBCs: red blood cells, PCV: packed cell volume, Hb: hemoglobin, MCH: mean corpuscular hemoglobin, NEU: neutrophils, CRP: C-reactive protein, PCT: procalcitonin, A/G:

albumin/globulin ratio, MDR: multidrug-resistant, XDR: extensively drug-resistant, PDR: pan drug-resistant.

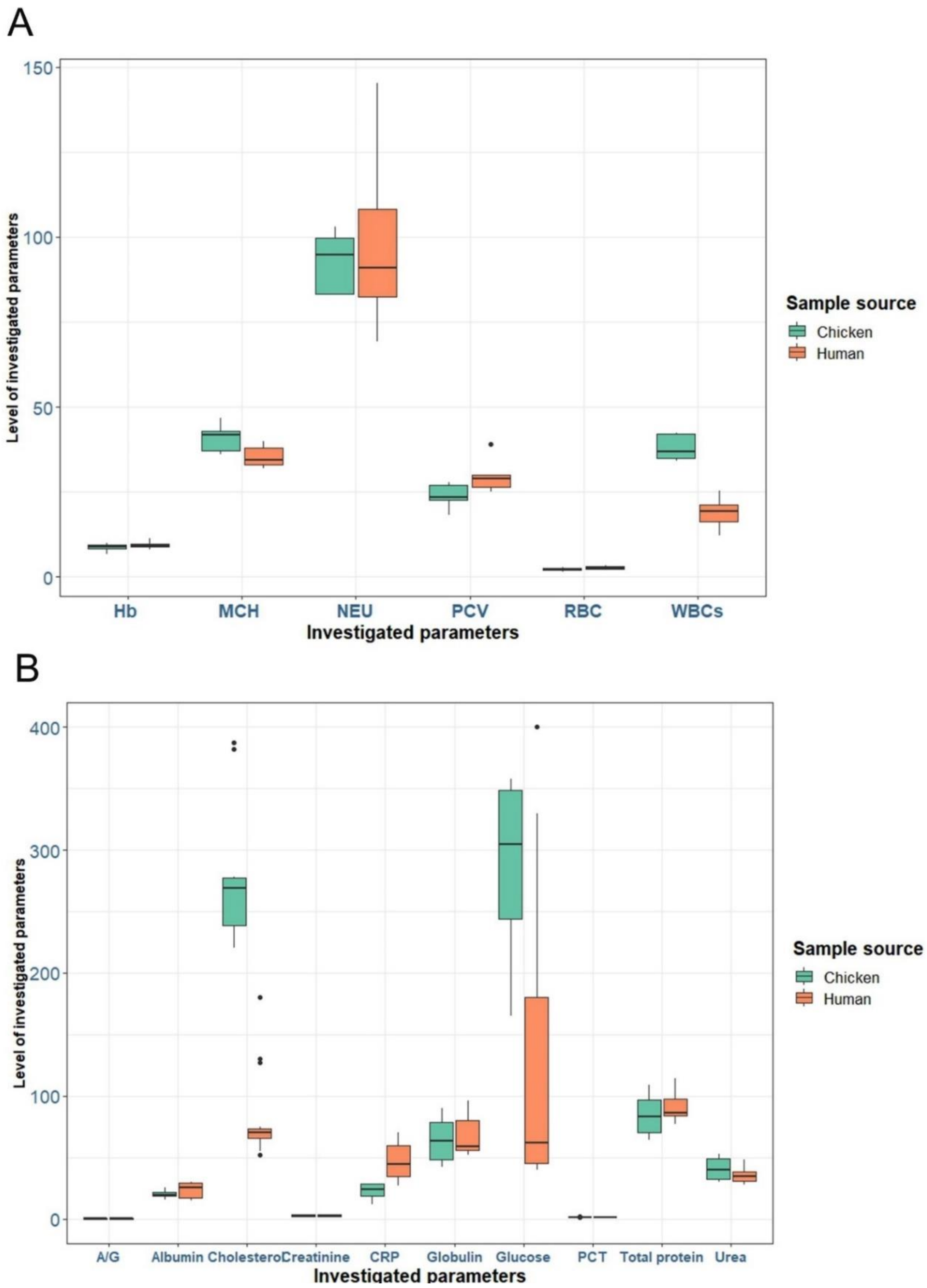

Supplementary Figure S5. Analysis of hematological and biochemical parameters among diseased chickens, and humans infected with *Acinetobacter baumannii*. WBCs: white blood cells,

RBCs: red blood cells, PCV: packed cell volume, Hb: hemoglobin, MCH: mean corpuscular hemoglobin, NEU: neutrophils, CRP: C-reactive protein, PCT: procalcitonin, A/G: albumin/globulin ratio.

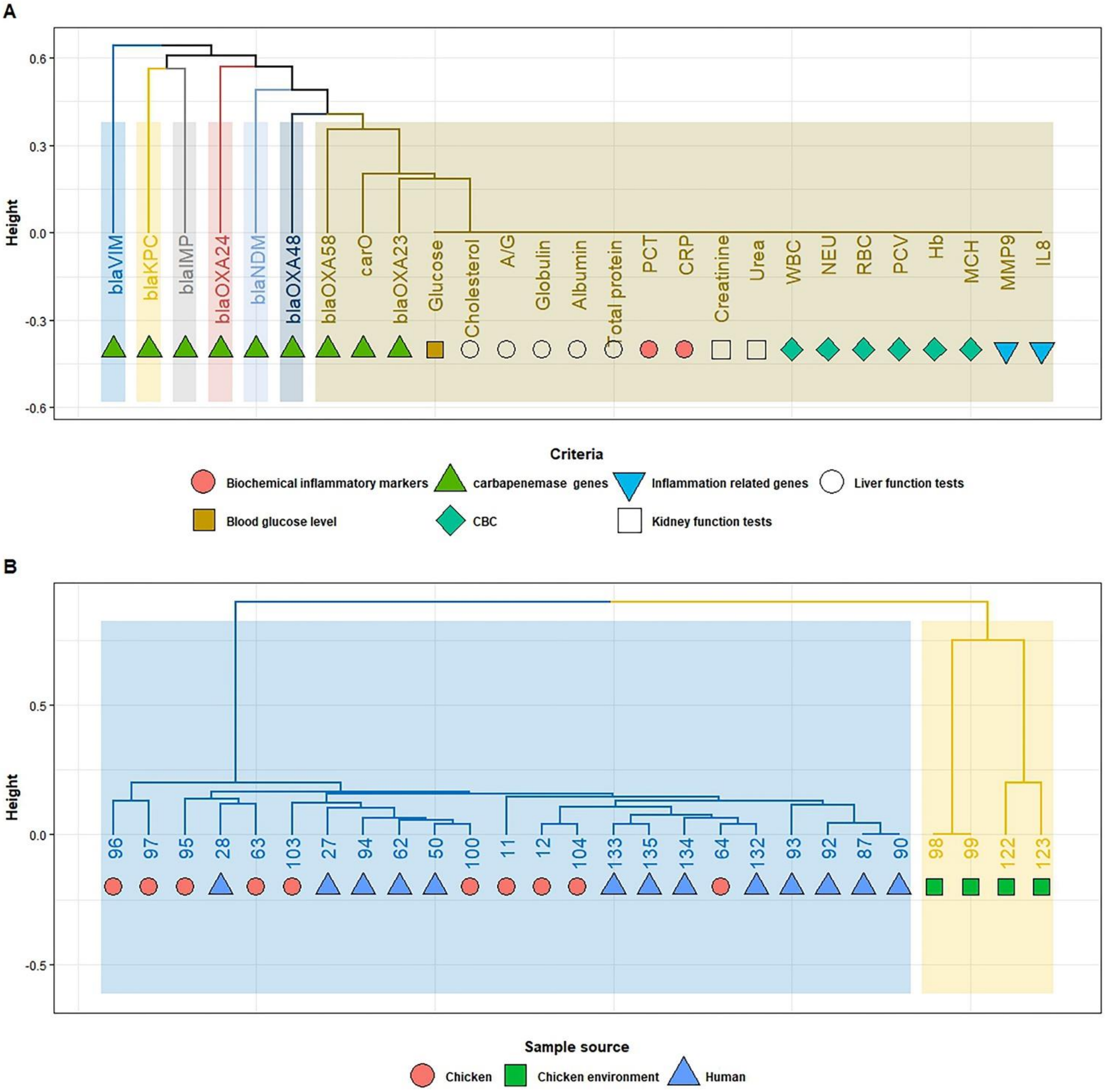

Supplementary Figure S6. Hierarchical clustering dendrogram revealing the binary distances among the investigated variables (A), and PDR *Acinetobacter baumannii* isolates (B) based on the frequency distribution of carbapenemase genes, hematological, and biochemical parameters, and the expression of *IL-8*, and *MMP-9* genes. The isolates are classified based on their sample source, which is presented in various colors and symbols. The X-axis refers to the binary distance scale. WBC: white blood cells, RBC: red blood cells, PCV: packed cell volume, Hb: hemoglobin, MCH: mean corpuscular hemoglobin, NEU: neutrophils, CRP: C-reactive protein, PCT: procalcitonin, A/G: albumin/globulin ratio.

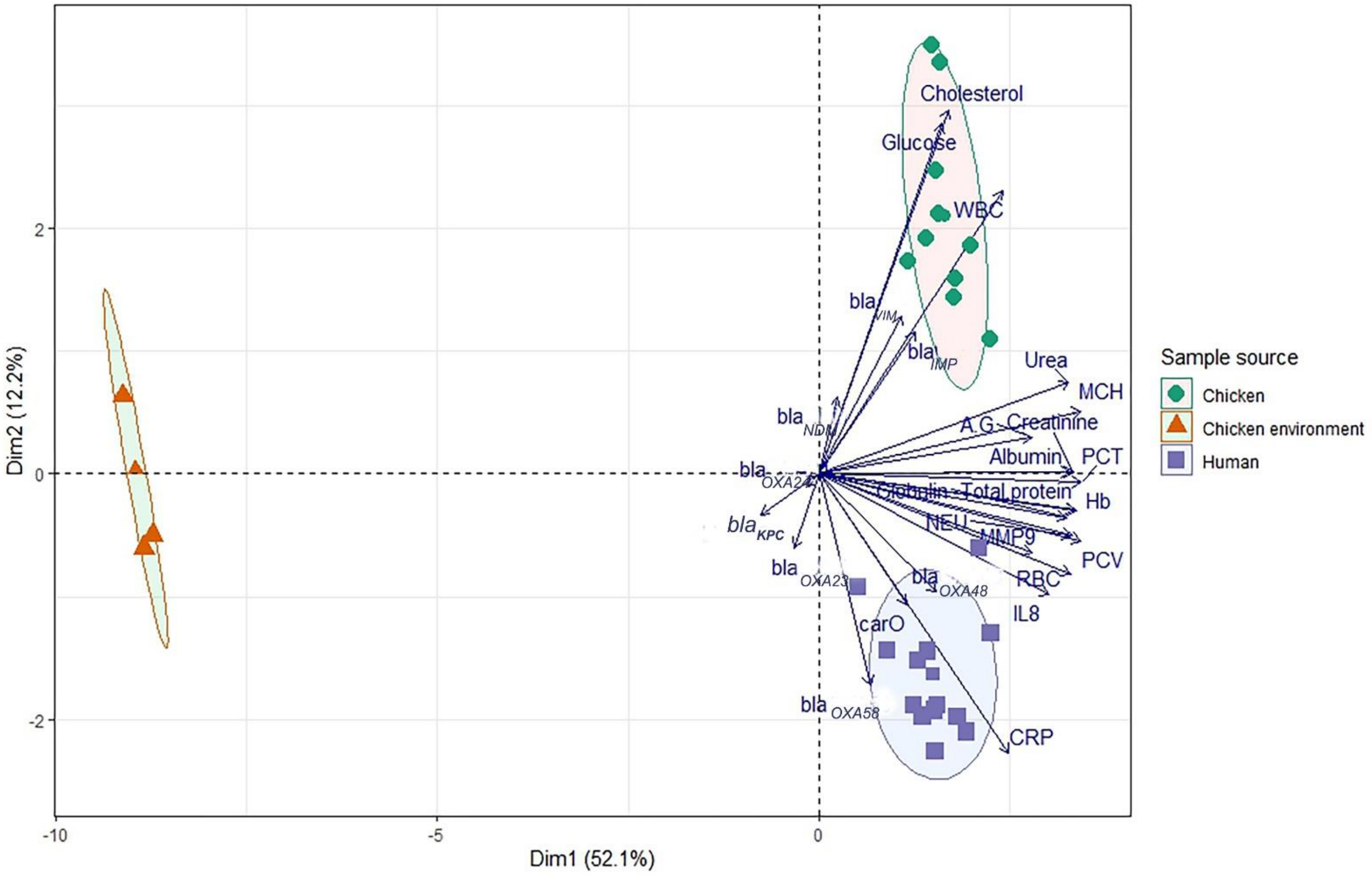

Supplementary Figure S7. PCA (Principal component analysis) biplot revealing the overall distribution of PDR *Acinetobacter baumannii* isolates from diseased chickens and hospitalized patients based on the frequency distribution of carbapenemase genes, hematological, and biochemical parameters, and the expression of *IL-8* and *MMP-9* genes. Each dot refers to one isolate, and the arrows refer to the association of each variable with either dimension 1 or 2. WBC: white blood cells, RBC: red blood cells, PCV: packed cell volume, Hb: hemoglobin, MCH: mean corpuscular hemoglobin, NEU: neutrophils, CRP: C-reactive protein, PCT: procalcitonin, A/G: albumin/globulin ratio
